# Quantifying the success of prey crypsis, aposematism, and evasiveness in avoiding predator attack

**DOI:** 10.1002/ecy.70248

**Published:** 2025-11-19

**Authors:** Daniel Linke, Jacqueline Hernandez Mejia, Valery N. P. Eche Navarro, Prapti Gohil, César Ramírez García, Letty Salinas, Marianne Elias, Pável Matos‐Maraví

**Affiliations:** ^1^ Biology Centre CAS (Czech Academy of Sciences) Institute of Entomology České Budějovice Czechia; ^2^ Department of Zoology, Faculty of Science University of South Bohemia České Budějovice Czechia; ^3^ Museum of Natural History and Facultad de Ciencias Biológicas of the Universidad Nacional Mayor de San Marcos Lima Peru; ^4^ Independent Researcher Lima Peru; ^5^ Instituto de Investigación Biológica de las Cordilleras Orientales Tarapoto Peru; ^6^ Institut de Systématique, Evolution, Biodiversité, Muséum National d'Histoire Naturelle, CNRS, Sorbonne Université, EPHE, Université des Antilles Paris France; ^7^ Smithsonian Tropical Research Institute Colón Panama; ^8^ Center for Interdisciplinary Research in Biology, CNRS, INSERM, Collège de France PSL University Paris France

**Keywords:** antipredator defenses, behavioral experiments, butterfly, insectivorous birds, predation sequence, predator–prey interaction

## Abstract

Antipredator defenses typically act at distinct stages of the predation sequence—encounter, identification, approach, and subjugation. However, their effectiveness has rarely been quantified and compared simultaneously in wild predator–prey systems. We conducted a study in Peru, where we installed aviaries at two localities and recorded the responses of wild avian predators to three types of antipredator defenses—crypsis, aposematism, and evasiveness—expressed by three butterfly prey types. The study included both immature and adult birds from forest and urban environments, representing the present community of insectivorous birds. We tested the theoretical expectations that cryptic butterflies (Nymphalidae: Euptychiina) were rarely detected, aposematic *Heliconius* (Nymphalidae: Heliconiinae) were often sighted but seldom attacked, and evasive *Spicauda* (Hesperiidae: Eudaminae) were frequently detected and attacked but evaded capture at higher rates. Despite these distinct defensive strategies, mortality rates among prey types were largely similar, but predator life stage strongly influenced defense effectiveness, with immature birds tending to attack *Heliconius* more frequently. Additionally, predator family influenced predation patterns, with more skilled insectivores (e.g., Vireonidae) showing higher capture success against defended prey. These findings illuminate the evolutionary pressures that shape predator behavior and prey defenses in tropical ecosystems. The similar mortality rates underscore the adaptive value of these defenses, which collectively distribute the total predation pressure across prey species.

## INTRODUCTION

Selective pressures associated with predation have shaped a wide range of defensive strategies in prey species. Antipredator mechanisms are often most successful at deterring a specific sequential stage of the predation sequence (Endler, 1991; Ruxton et al., 2018). For example, crypsis reduces the likelihood of encountering and detection, whereas aposematism relies on conspicuous signals that advertise unprofitability to deter attacks by predators that have learned the warning signals. By contrast, evasive ability is a defense strategy that impedes subjugation and consumption once predators are engaged in pursuing prey. Understanding how wild predators interact with and respond to the occurrence of multiple defended prey, and how the effectiveness of antipredator defenses is shaped by the community composition of predators, provides crucial insights into the evolutionary pressures affecting prey phenotypic diversity.

Among prey species exhibiting a broad arsenal of defensive strategies, butterflies possess antipredator defenses that operate on each stage of the predation sequence. Cryptic butterflies, for example, typically reduce predator encounters and detection through background matching (Feltmate & Williams, 1989; Pembury Smith & Ruxton, 2021; Pinheiro & Campos, 2019; Vallin et al., 2006), which can be assessed in the wild by wing damage, though the true predation risk of cryptic prey remains unclear (Molleman et al., 2020). Unpalatable butterflies, such as Heliconiini and Ithomiini (Mallet & Gilbert, 1995), advertise their unprofitability through aposematic cues that avian predators learn to avoid (Arias et al., 2016; Chouteau et al., 2016; Chouteau et al., 2019; Willmott et al., 2017), but these experiments are usually limited by not assaying taste rejection after attack (Mappes et al., 2014; Seymoure et al., 2018). Finally, evasive flight behavior (Chai, 1986; Pinheiro & Freitas, 2014; Srygley & Chai, 1990) and deflective structures may prevent deadly attacks on butterflies after being spotted (Chotard et al., 2022). Evasiveness triggering predator learning and being used during prey identification is theoretically possible (Ruxton et al., 2004), and recent studies have shown that predators learn to attack nonevasive butterfly models (Linke et al., 2022; Páez et al., 2021).

No antipredator defense offers complete protection against predation, especially in ecosystems harboring a large diversity of predator species. However, our understanding of how wild predators respond to simultaneously encountered prey with diverse defense strategies is limited (observations of wild hunting Jacamars—Pinheiro & Campos, 2019; or studies using wild predators but dummy prey—Guerra et al., 2024; Dell'aglio et al., 2016). By conducting behavioral experiments in aviaries with wild insectivorous birds, we aim to understand how predators respond when multiple prey species with distinct antipredator defenses are presented simultaneously, as well as how effective different antipredator defenses are when avoiding predation.

**FIGURE 1:**
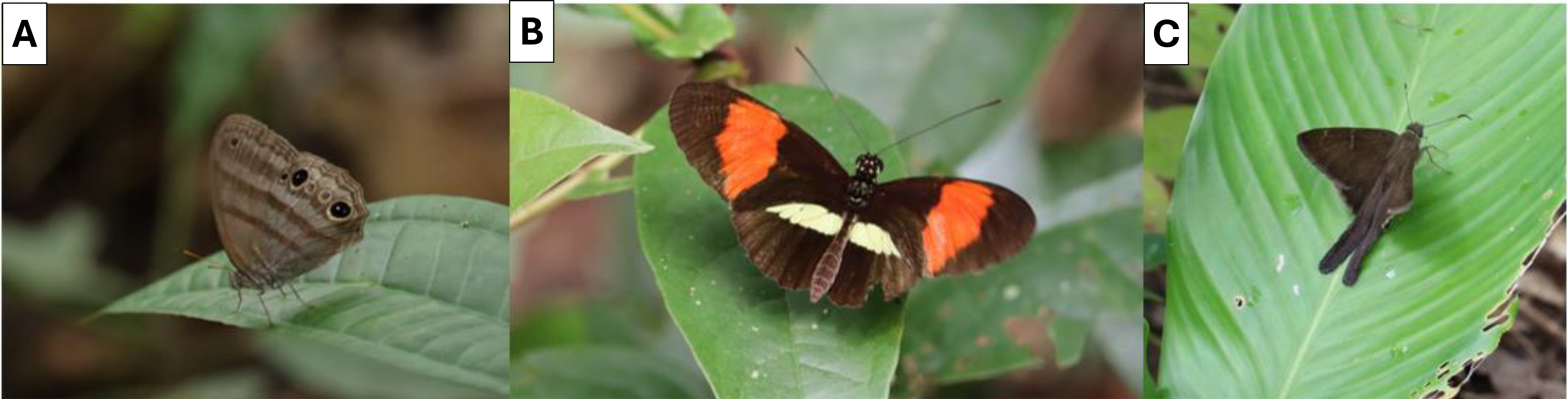
Examples of butterflies in their native environment, used in the aviary experiments to assess responses of birds to different antipredation strategies, (A) The cryptic *Cissia penelope* (Fabricius, 1775), (B) The aposematic *Heliconius erato* (Linnaeus, 1758), and (C) The evasive *Spicauda simplicius* (Stoll, 1790) (photos not scaled). Butterfly photos by Daniel Linke.

The prey that we study are butterflies with antipredator defenses believed to act at each major stage of the predation sequence: (1) Euptychiina (Nymphalidae: Satyrinae), which likely relies on crypsis to avoid being detected, (2) *Heliconius* (Nymphalidae: Heliconiinae), which likely relies on aposematism and Müllerian mimicry to avoid being identified as profitable prey, and (3) *Spicauda* (Hesperiidae: Eudaminae), which likely relies on evasive behavior to avoid being caught (Figure 1). Here, we investigate how predator responses vary by life stage, diet, and taxonomic family.

**FIGURE 2:**
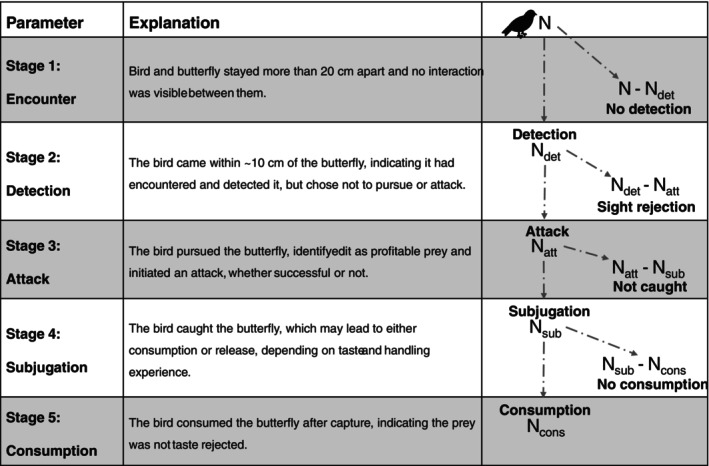
Stages of the predator–prey interactions observed during the experiment, including a dichotomous flowchart depicting the progression of birds through the predation sequence. Figure created by Daniel Linke using a bird silhouette icon from Microsoft PowerPoint Icons (used under Microsoft Office licensing terms).

Based on the predator–prey interaction theory, we test the following hypotheses along the predation sequence (Figure 2): (1) The probability to remain undetected will be highest for cryptic butterflies (i.e., the dull brown coloration of Euptychiina and *Spicauda*) while conspicuous butterflies (i.e., the aposematic *Heliconius*) have higher probabilities to be detected by birds. (2) Butterflies that advertise their unprofitability through conspicuous color patterns (i.e., *Heliconius*) are more likely to be rejected without attack by birds upon identification. Species lacking learned visual cues associated with unprofitability will be more likely to get attacked outright (e.g., the presumably slow flying, dull colored, and palatable Euptychiina). (3) Evasive butterflies (i.e., *Spicauda*) will be more likely to escape predator attacks, compared to slow‐flying butterflies (i.e., *Heliconius* or Euptychiina). (4) Structural or chemical defenses (sturdy wings of unpalatable *Heliconius* or the hindwing tails of *Spicauda*) will result in lower mortality rates by being released with nonfatal damage.

## METHODS

### Study location

The experiments were carried out during the rainy season of 2021/2022 (October–February) and the dry season of 2022 (June–September). The two study locations were (1) within the city limits of Tarapoto, Peru (6.47° S, 76.35°W, ~400 m above sea level [asl]), and (2) within a privately protected area, the Urahuasha sector (6°27′ S 76°20′ W, ~700 m asl), near the external border of the “Área de Conservación Regional Cordillera Escalera”, San Martin, Peru. The first study site was characterized by significant human intervention, and the second site was characterized by secondary growth forest (~50 years old) with very limited human influence.

### Birds

We installed dull‐colored mist nets near pathways in scrub habitats, secondary forests, forest edges, and hill crests at both study sites (Appendix S1: Section S1: Tables S1 and S2). Nets were opened from ~7 AM and checked hourly until dusk (~5 PM). Captured birds were identified to species, their age determined (immature or adult), and photographed. Immature birds were identified by plumage and commissure color. Birds selected for behavioral experiments were known to feed on butterflies (Pinheiro & Cintra, 2017), classified as insectivorous (Egg et al., 2010), or their insectivorous diet was confirmed by local experts. Birds were categorized as fully insectivorous or omnivorous/insectivorous. All other birds were marked and released. Experimental birds were kept in captivity for up to 30 h at the urban site and 12 h at the forest site, with constant access to water and local papaya.

### Butterflies

Every experiment included three butterfly prey types employing different defensive strategies. With respect to the first hypothesis involving cryptic prey, we studied medium‐sized Euptychiina, consisting mainly of *Cissia penelope* and related, syntopic species in the genera *Cissia*, *Magneuptychia*, and *Yphthimoides. Heliconius erato* was utilized as an unpalatable, aposematic prey, and when unavailable, we used the co‐mimetic *H. melpomene*. Lastly, as a representative of prey relying on evasive flight to avoid predation, we studied *Spicauda simplicius*, and when unavailable, we used the morphologically similar and syntopic *S. teleus*, *S. tanna*, and *S. procne* (Figure 1). All species within each study phenotype shared similar morphologies, behaviors, and co‐occurred in the same microhabitats.

Butterflies were caught with entomological nets around the study sites and identified to the species level, with Euptychiines sometimes only to the genus level. They were kept alive in mesh cages (50 × 50 × 50 cm) with fake flowers filled with sugar water. Each butterfly was used in up to three predation experiments or for a maximum of 48 h to prevent fatigue. Afterward, they were sacrificed and stored in silica gel for morphological identification.

### Behavioral experiments in aviaries

At each study location and in an open space to obtain similar luminosity, we constructed an experimental aviary (*H* × *W* × *L* = 4 × 2 × 4 m) made of green garden mesh and plumbing tubes (Appendix S1: Section S2: Figure S1). We installed bamboo perches for the birds to rest on at ~50 cm below the aviary ceiling. We installed two video cameras (GoPro HERO6, San Mateo, USA; set to 1080p, 60 fps, with the wide lens setting) on the two front sides of the aviary at ~1 m height, allowing full video coverage of the aviary. The experiments were conducted from ~7 AM to ~6 PM depending on the availability of birds and butterflies.

The behavioral experiments involved simultaneously presenting the three butterfly types to a bird in an aviary while recording the interactions between the predator and the prey. The bird was first starved for 60 min, and then acclimatized in the aviary for 30 min. Then, the butterflies were released, and the cameras recorded the experiment for 60 min, while avoiding any human presence to reduce stress. At the end, any remaining butterflies were recaptured, and the ground was surveyed for any detached wings. After cutting two tail feathers for identification, the bird was released before dark under favorable weather conditions. Recaught birds were not used again. The experiment was invalid if there were disturbances like sudden rainfall, the appearance of cats or monkeys, or signs of bird fatigue (e.g., lack of movement or erratic flight).

### Video processing

We aligned the two cameras to allow for the simultaneous observation of both cameras, using DaVinci Resolve 18 (Blackmagic Design, Melbourne, Australia). We noted the behavioral responses of birds and butterflies as timestamps (Figure 2), recording the start and end of stages in the predation sequence: encounter, identification, approach, subjugation, and consumption. Each bird–butterfly combination was assigned the highest observed stage in the predation sequence. Birds that did not interact with any butterfly throughout the experiment, that is, no pursuit, sight‐rejection, or attack, were excluded from the analysis. We also recorded the approximate time the bird and each butterfly were present at two heights in the aviary: ground level, 0–200 cm, and ceiling level, 200–400 cm.

### Chance of predator–prey encounter

To estimate the encounter probability, we calculated the co‐occurrence rate of the bird and each butterfly, using:

(1) $$\mathrm{Co}={\text{Bird}}_{\mathrm{low}}\times {\text{Butterfly}}_{\mathrm{low}}+{\text{Bird}}_{\text{high}}\times {\text{Butterfly}}_{\text{high}},$$

where the first term is the proportion of experimental time the bird and a butterfly co‐occurred at the ground level (0–200 cm) and the second term is the proportion of time they co‐occurred at the ceiling level (200–400 cm). The Co index ranges from 0 to 1, where 0 indicates no vertical overlap between a bird and a butterfly (i.e., no chance of encounter), and 1 indicates complete overlap (i.e., high chance of encounter). For example, if a bird spends 80% of its time in the upper stratum (200–400 cm) and 20% in the lower stratum (0–200 cm), and a butterfly spends 80% of its time in the lower and 20% in the upper stratum, the Co index would be: Co = (0.20 × 0.80) + (0.80 × 0.20) = 0.32.

### Predictors of bird responses to prey phenotypes: Logistic regressions

We used generalized linear mixed models (GLMMs) with a binomial distribution (via the *glmer* function from the lme4 R package v.1.1‐37; Bates et al., 2015) to identify bird responses (sight rejection or attacking) in relation to prey type, bird age (immature or adult), and diet (insectivorous or mixed). Bird identity was included as a random effect to account for individual variability. Each bird–butterfly interaction was transformed into a binary outcome based on the highest observed behavioral stage (Figure 2). These two separate models were used to compare sight rejection and attack behaviors:

(2a) $$\text{logit}\left(P\left(Y_{\text{sight}-\text{reject}}=1\right)\right)=\beta_{0}+\beta_{\text{butterfly}\_\text{type}}\times X_{\text{butterfly type}}+\beta_{\mathrm{age}}\times X_{\mathrm{age}}+\beta_{\text{diet}}\times X_{\text{diet}}+u_{\text{birdID}},$$

(2b) $$\text{logit}\left(P\left(Y_{\text{attack}}=1\right)\right)=\beta_{0}+\beta_{\text{butterfly}\_\text{type}}\times X_{\text{butterfly type}}+\beta_{\mathrm{age}}\times X_{\mathrm{age}}+\beta_{\text{diet}}\times X_{\text{diet}}+u_{\text{birdID}},$$

where *Y* is the binary outcome of a butterfly being sight rejected or attacked by a bird; $\beta_{0}$ is the general tendency to sight reject or attack; $\beta_{\text{butterfly}\_\text{type}}\times X_{\text{butterfly type}}$, $\beta_{\mathrm{age}}\times X_{\mathrm{age}}$, and $\beta_{\text{diet}}\times X_{\text{diet}}$ represent the effects of butterfly type, age, and diet, respectively; and $u_{\text{birdID}}$ accounts for variation among individual birds. For the sight‐rejection model (2a), the response variable was coded as 1 for sight rejection (*N*_det_ − *N*_att_, that is, by being ~10 cm close to the butterfly without attacking behavior; *N*_det_ is the number of birds that detected a butterfly, and *N*_att_ is the number of birds that attacked the butterfly; Figure 2) and 0 for attacking behavior (*N*_att_). In the attacking model (2b), the response variable was coded as 1 for active attacking (*N*_att_), and 0 for all other responses (*N*_det_–*N*_att_ and *N*–*N*_det_, i.e., sigh rejection or no interaction at all). Pairwise comparisons were facilitated using the *emmeans* function from the *emmeans* R package v. 1.10.0 (Lenth, 2025). Repeated use of individual butterflies was not modeled as a random effect, as individual bird behavior contributes more to the outcome than variation among butterflies, and adding butterfly identity would substantially increase model complexity.

### Probabilities of attack deterrence at different stages in the predation sequence

We used log‐likelihood tests to compare the probabilities of prey to (1) remain undetected, (2) get sight‐rejected upon detection, (3a) get attacked, (3b) avoid capture during a predator attack, and (4) survive a predator attack. The tests were performed using the birds that detected at least one butterfly prey (any interaction: *N*–*N*_det_), that is, birds that were ~10 cm close to the prey and birds that showed active attacking behavior (Figure 2). Further tests were also performed using the data subset by predator age (immature or adult), season (dry or wet), and habitat (forest or urban). We calculated the log‐likelihood of models depicting the number of events within each predation sequence stage for a particular prey phenotype compared to others, following the method implemented in Mérot et al. (2015), Willmott et al. (2017), and Páez et al. (2021):

(3) $$\;\log_{10}\left(L\right)=\sum_{i}\left[a_{i}\log_{10}\left(P_{i}\right)+\left(N-a_{i}\right)\log_{10}\left(1-P_{i}\right)\right]+K,$$

where *i* represents the prey type (Euptychiina, *Heliconius*, or *Spicauda*); *N* denotes the total count of the prey presented (i.e., number of birds), *a*_*i*_ is the count of interactions with the prey type *i*; *P*_*i*_ is the attack probability for each prey type *i*; and *K* is a constant that cancels out when comparing models. For each stage, we compared five competing models: All prey having different probabilities of interaction (“all different”), the same probabilities (“all equal”), and two of the prey phenotypes having the same probability but different from the third phenotype (“Euptychiina different,” “*Heliconius* different,” and “*Spicauda* different”). The model with the lowest AICc value was selected as the best model, while any model within 2 units of AICc was not rejected. We calculated Akaike weights following Burnham and Anderson (2002) to compare differences among the five competing models for each predation stage.

### Description of the likelihood‐based model tests for each stage in the predation sequence

*(1) Probability to remain undetected*: We compared the sum of occasions for each prey phenotype where the bird and butterfly did not interact (*N*–*N*_det_, i.e., prey was not encountered or detected) to the total number of tested birds that did enter the predation sequence for at least one butterfly (*N*).

In addition to the likelihood‐based comparison, we compared Co index values between butterflies using pairwise Wilcoxon signed‐rank test with Bonferroni correction (for multiple comparisons) as the normal distribution of the data was rejected (Shapiro–Wilk test: *N* = 393, *W* = 0.897, *p* = 1.355 × 10^−15^).

*(2) Probability of being rejected upon detection*: We compared the sum of events for each prey phenotype where the bird was close to the butterfly (~10 cm) but did not engage in any attacking behavior (*N*_det_–*N*_att_), in comparison to all birds that did enter the predation sequence for at least one butterfly (*N*). We assumed that the birds in spatial proximity to a butterfly were able to spot the prey but decided not to pursue any attacking behavior (sight rejection).

*(3) Targeting probability and capture avoidance*: This involves two steps, (3a) the chance of getting attacked in the first place and (3b) the ability to avoid being caught when attacked. First, (3a) we compared the sum of events where the butterfly was actively attacked by the bird depending on the butterfly type, regardless of the outcome (*N*_att_), in comparison to all birds that did enter the predation sequence for at least one butterfly (*N*). Second, (3b), to determine whether prey phenotypes differ in their ability to avoid being captured, we additionally computed the sum of events where the butterfly was not captured (*N*_att_–*N*_sub_) in comparison to the sum of birds showing active attacking behavior to each prey phenotype (*N*_att_).

Lastly, we determined for each bird and butterfly combination the time for the first instance of attacking behavior (no attack, equals 60 min) to compare any preference in engaging to pursue different butterfly types. As a normal distribution of the data was rejected (Shapiro–Wilk test: *N* = 217, *W* = 0.747, *p* = 2.2 × 10^−16^), we performed pairwise Wilcoxon signed‐rank tests with Bonferroni correction (for multiple comparisons) to compare initial attack time distributions between butterflies.

*(4) Probability to survive upon predator attack*: We compared the sum of failing to kill (*N*_att_–*N*_cons_) for each prey phenotype in comparison to the number of actively attacked butterflies per phenotype (*N*_att_).

Statistics (except log‐likelihood tests, which were implemented in a spreadsheet) were done using R v. 4.3.0 (R Core Team, 2023). Graphics were generated using the R package *ggplot2* v. 3.4.3 (Wickham, 2016).

## RESULTS

We performed 262 experiments, of which 216 were deemed valid. There were 65 valid experiments in the urban environment and 151 experiments in the forested habitat, of which 70 were performed in the wet season and 81 in the dry season. Due to time and logistical constraints, we were not able to carry out experiments in the urban environment during the dry season.

We studied 41 bird species from 13 families. The number of insectivorous species was higher in the forested habitat than in the urban environment (urban/wet season = 12 species; forest/wet season = 19 species; forest/dry season = 23 species) (list of experimental bird species given in Appendix S1: Section S3: Table S3). Additional details on captured bird species and photographs are available in field guides (Navarro et al., 2023, Hernández Mejía et al. 2025). In the urban area, 68% of birds were adults and 32% were immatures, while in the forest, 29% were adults and 71% were immatures during the wet season, and 16% were adults and 84% were immatures in the dry season (species‐level data of experimental birds in Appendix S1: Section S3: Table S3).

### General differences between prey types and bird families

Of birds that sight rejected or attacked at least one butterfly, nearly 70% did not interact with Euptychiines (*N*–*N*_det_), whereas this proportion was notably lower for *Heliconius* (34%) and *Spicauda* (18%). Sight rejection (*N*_det_–*N*_att_) was most prevalent toward *Heliconius*, with almost 50% of birds exhibiting this response, followed by *Spicauda* (18%) and Euptychiines (9%). *Spicauda* was most frequently attacked by birds (*N*_att_, 40%), while attack rates for Euptychiines and *Heliconius* were comparable at 21% and 18%, respectively. Only 6% of all birds caught *Heliconius* (i.e., *N*_sub_, killing or releasing it afterward), compared to 16% for *Spicauda* and 12% for Euptychiines (plot for diet and bird family in Appendix S1: Section S4: Figure S2A). Overall mortality rates (*N*_cons_) were similar across all three prey phenotypes, with Euptychiines at 7%, *Heliconius* at 4%, and *Spicauda* at 9%.

**FIGURE 3:**
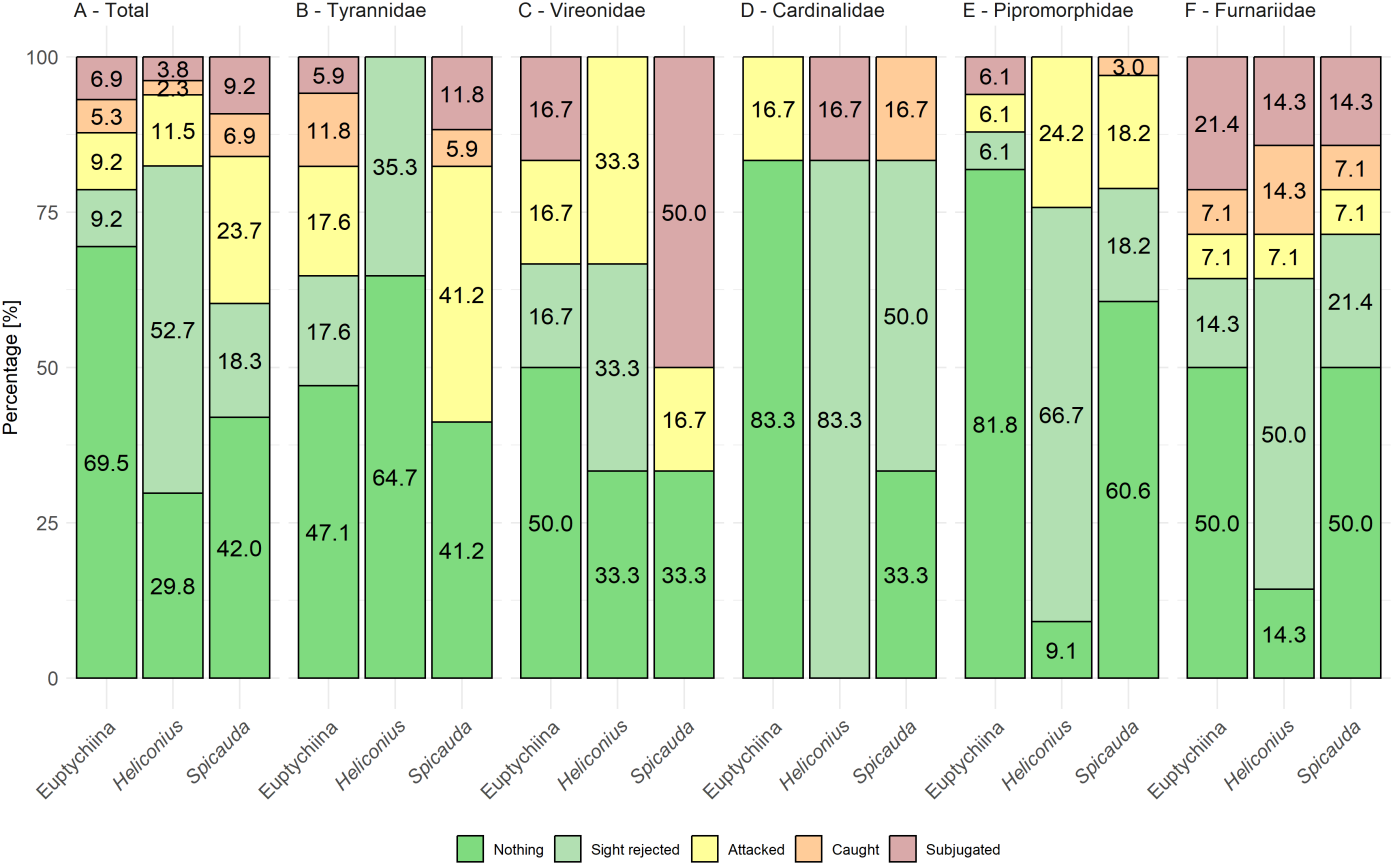
Comparison of predation outcomes depending on the main bird family and butterfly type. (A) Predation outcomes across all tested birds, (B) Outcomes for Tyrannidae, representing agile aerial insectivores, (C) Outcomes for Vireonidae specialized foliage gleaning insectivores, (D) Cardinalidae, which are largely granivorous or frugivorous, (E) Pipromorphidae, small insectivorous birds, and (F) Furnariidae, insectivorous birds foraging along tree trunks and the ground.

Among bird families, Furnariidae were most effective in attacking Euptychiines, with 21% successfully catching them, while Thraupidae were the least effective, with only 8% exhibiting some interaction (i.e., at least sight rejection) (Figure 3, Appendix S1: Section S4: Figure S2I,E). Most bird families showed similar probabilities of sight rejecting *Heliconius*, ranging between 30% and 50% (Figure 3, Appendix S1: Section S4: Figure S2D–K), while Pipromorphidae and Cardinalidae reached even higher levels at 66 and 83%, respectively. Tyrannidae and Vireonidae, two highly insectivorous families and skilled flycatcher hunters, were the only bird families that did not sight reject *Spicauda*, with Vireonidae displaying the highest success in capturing them (Figure 3, Appendix S1: Section S4: Figure S2D,F).

### Predictors of bird behaviors to prey phenotypes: Logistic regressions

The GLMM for sight rejection behavior included 208 observations across 131 individual birds and revealed that birds were more likely to sight reject *Heliconius* than both Euptychiines (emmeans: *z* = 7.33, *p* = 1.34 × 10^−11^) and *Spicauda* (*z* = −6.92, *p* = 6.96 × 10^−13^). No significant differences were found between Euptychiines and *Spicauda* (*z* = 0.15, *p* = 0.99). Bird age (*z* = −0.71, *p* = 0.48) or diet (*z* = −0.33, *p* = 0.74) did not affect sight rejection behavior. Estimated sight rejection probabilities were near 100% for *Heliconius* (probability = 0.99938), and extremely low for *Spicauda* (prob. = 0.00056) and Euptychiines (prob. = 0.00067). Rejection probabilities were lower for adult birds (prob. = 0.053) compared to immature ones (prob. = 0.114). Differences between diet classes were minor (insectivorous: prob. = 0.065, other: prob. = 0.093). Random intercept variance indicated substantial individual‐level variability (σ^2^ = 304.8, SD = 17.46).

The GLMM for attack behavior included 393 observations across 131 individual birds and showed that birds were significantly more likely to attack *Spicauda* than Euptychiines (emmeans: *z* = −3.36, *p* = 0.0023) or *Heliconius* (*z* = −4.04 *p* = 0.0002). No significant differences were observed between *Heliconius* and Euptychiines (*z* = 0.83, *p* = 0.686). Estimated attack probabilities were highest for *Spicauda* (prob. = 0.377) and considerably lower for Euptychiines (prob. = 0.176) and *Heliconius* (prob. = 0.139). Bird age (*z* = 0.50, *p* = 0.617) or diet (*z* = −0.387, *p* = 0.698) had no significant effect on attack probabilities. Individual variation in attacking behavior was low (σ^2^ = 0.88, SD = 0.94).

The full model outputs and additional GLMM models including bird family, habitat, and season can be found in Appendix S1: Section S5. Notably, regarding sight rejection behavior, specialized aerial predators like Vireonidae and Tyrannidae had lower expected sight rejection probabilities (prob. = 0.212 and 0.276, respectively) than bird families more reliant on grains and fruits (Cardinalidae: prob. = 0.879, Turdidae: prob. = 0.699), while other specialized families, that is, Thamnophilidae (ant birds) or Furnariidae (tree creepers), ranked in between (prob. = 0.473 and 0.570, respectively) (Figure 3). Season and habitat did not significantly influence sight rejections (season: *z* = 0.701, *p* = 0482; habitat: *z* = −0.974, *p* = 0.330), although sight rejections tended to be more likely during the dry (prob. = 0.614) compared to the wet season (prob. = 0.501) and more likely in the forested area dominated by immatures (prob. = 0.672) compared to the urban area harboring mostly adults (prob. = 0.439). Attacking behavior did not significantly differ between families, but was more likely in specialized aerial foragers, that is, Tyrannidae and Vireonidae (prob. = 0.365 and 0.432, respectively) than in families more reliant on grains or fruits (i.e., Cardinalidae prob. = 0.083) (Figure 3). Season (*z* = −0.976, *p* = 0.329) and habitat (*z* = 1.790, *p* = 0.073) did not show significant effects, although slightly higher attack probabilities were observed during the wet (prob. = 0.238) compared to the dry season (prob. = 0.171) and attacks were slightly less than three times more likely in the forest (prob. = 0.307) compared to the urban habitat (prob. = 0.127).

### Likelihood‐based comparisons along the predator–prey interactions

*(1) Probability to remain undetected*: Across all bird families in different habitats and seasons, there was a general preference for foraging near the ceiling level (Appendix S1: Section S6: Figure: S7A). Vireonidae (nine individuals and two species) spent on average only 14% of their time near the ground, whereas antbirds (Thamnophilidae, 23 individuals, and 9 species) spent most of the time (66%) foraging near the ground. Butterflies, on average, differed in their stratum selection (Appendix S1: Section S6: Figure S7B). Euptychiines tended to occur most of the time (55%) near the ground, whereas *Spicauda* and *Heliconius* spent, on average, 78% and 83% of their time, respectively, in the upper stratum.

**FIGURE 4:**
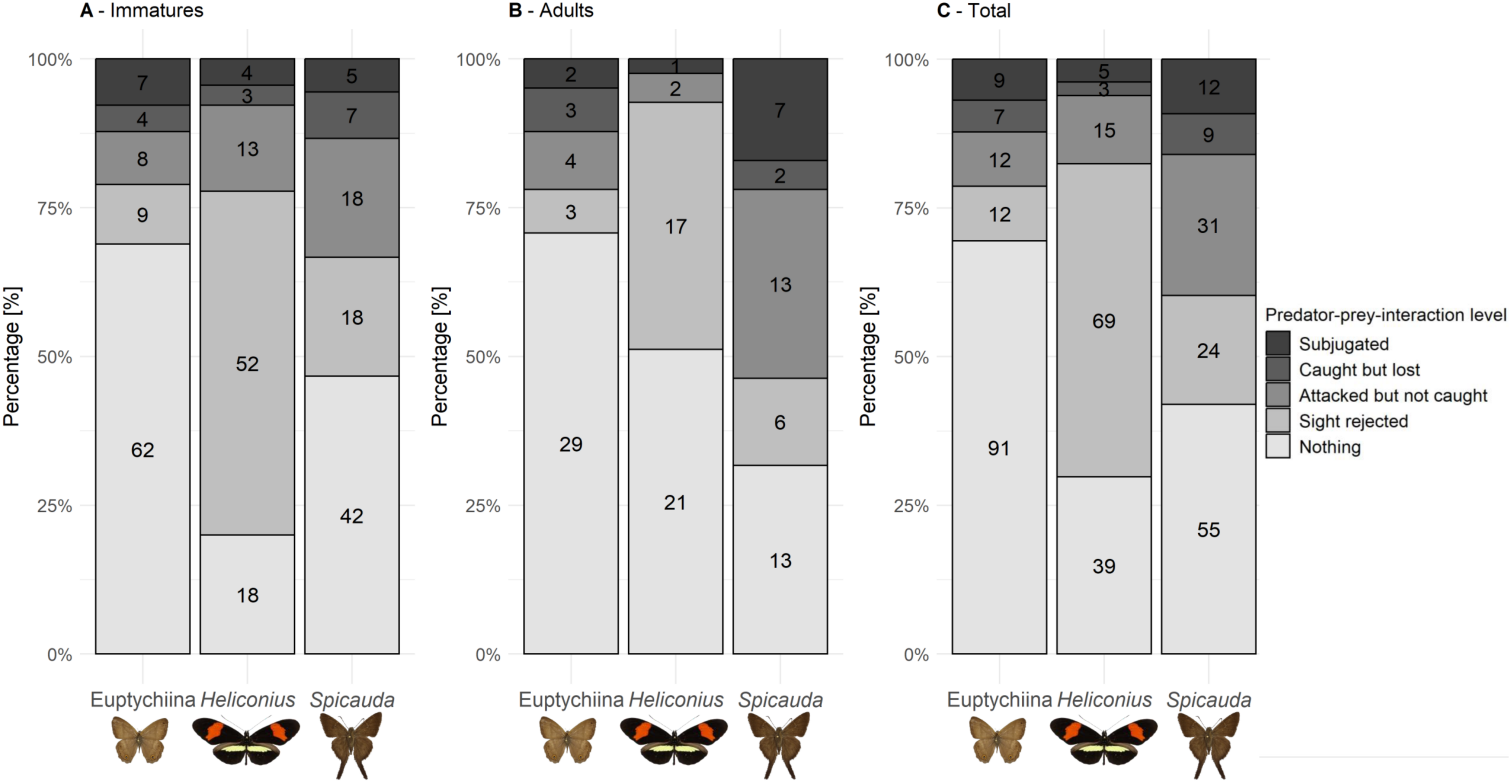
Comparison of predation outcomes (no interaction, sight rejected, attacked, caught, or subjugated) depending on the butterfly type (Euptychiines, *Heliconius*, and *Spicauda*) and depending on the age of experimental birds (adult and immature). Numbers inside bars represent the number of birds showing this interaction. (A) Distribution for all immature birds, (B) distribution for all adult birds, and (C) distribution of all predator–prey interactions, including all valid experiments. Butterfly pictures taken by Daniel Linke, photos in scale.

Our likelihood‐based model tests supported the “Euptychiines different” scenario (Akaike weight: 0.51; Figure 4, Table 1), which suggests that birds were overall least likely to encounter Euptychiines compared to *Heliconius* and *Spicauda*. Additionally, the “all different” scenario was the second‐best model (ΔAICc = 0.18, Akaike weight: 0.47), with birds most likely to encounter *Heliconius*. For adult birds, “Euptychiines different” and “*Spicauda* different” had almost equal Akaike weights (0.32 and 0.31, respectively), both scenarios describing that birds were least likely to encounter Euptychiines and most likely *Spicauda*. However, the “all equal” model (no difference among butterfly species) also had substantial support (Akaike weight: 0.22; ΔAICc = 0.69). For immature birds, “all different” was the best model, with Euptychiines the least and *Heliconius* the most likely to be encountered (Akaike weight: 0.67). Additionally, “*Heliconius* different” received substantial support (ΔAICc = 1.95, Akaike weight: 0.25).

**TABLE 1:** Likelihood‐based model tests of scenarios depicting the effectiveness of antipredator defenses by three butterfly prey types along the predation sequence.

Stage	Model	All difference	All equal	*Heliconius* difference	*Spicauda* difference	Euptychiina difference
(1) Remaining undetected	Total	**0.47** (0.18)	0.00 (15.45)	0.02 (6.98)	0.00 (16.58)	**0.51** (0.00)
	Immature	**0.67** (0.00)	0.00 (15.80)	**0.25** (1.95)	0.00 (17.77)	0.08 (4.34)
	Adult	**0.22** (0.69)	**0.11** (2.10)	0.04 (4.17)	**0.31** (0.03)	**0.32** (0.00)
(2) Rejection upon identification	Total	**0.50** (0.00)	0.00 (26.34)	**0.50** (0.02)	0.00 (25.15)	0.00 (13.10)
	Immature	**0.44** (0.49)	0.00 (20.40)	**0.56** (0.00)	0.00 (19.90)	0.00 (10.57)
	Adult	**0.24** (1.61)	0.06 (4.23)	**0.53** (0.00)	0.03 (5.59)	0.13 (2.78)
(3a) Targeting probability	Total	**0.25** (1.77)	0.04 (5.73)	0.07 (4.29)	**0.62** (0.00)	0.02 (6.70)
	Immature	0.10 (2.24)	**0.32** (0.00)	**0.14** (1.68)	**0.29** (0.21)	**0.16** (1.41)
	Adult	**0.36** (0.51)	0.02 (6.68)	0.15 (2.31)	**0.47** (0.00)	0.01 (8.30)
(3b) Capture avoidance	Total	0.09 (2.89)	**0.37** (0.00)	**0.16** (1.66)	**0.14** (1.88)	**0.24** (0.86)
	Immature	0.08 (3.30)	**0.39** (0.00)	**0.16** (1.76)	0.14 (2.01)	**0.22** (1.16)
	Adult	0.05 (4.37)	**0.46** (0.00)	0.15 (2.19)	0.16 (2.15)	**0.17** (1.98)
(4) Surviving attack	Total	0.06 (3.79)	**0.43** (0.00)	**0.16** (1.99)	**0.16** (1.97)	**0.19** (1.67)
	Immature	0.07 (3.36)	**0.40** (0.00)	**0.15** (2.00)	**0.16** (1.77)	**0.22** (1.18)
	Adult	0.05 (4.54)	**0.47** (0.00)	0.15 (2.25)	0.16 (2.19)	0.16 (2.12)

*Note*: Akaike weights for the total number of birds and bird age (immature vs. adult) are shown. Best models are marked in **bold and underlined** , while competing models (ΔAICc <2) are only **bold**. Values in brackets represent ΔAICc values compared to the best‐performing model. Sample sizes for (1) to (3b) are identical for all prey types: 131 for total, 90 for immature, and 41 for adults. For (4) and (5), sample sizes are different per prey type (depending on how many birds choose to attack each butterfly type): for the total, *Heliconius* has a sample size of 23, *Spicauda* of 52, and Euptychiines of 28, for immatures 20, 30, and 19; and adults 3, 22, and 9, respectively. Additional results for seasons and habitats can be found in Appendix S1: Section S7: Table S4.

**FIGURE 5:**
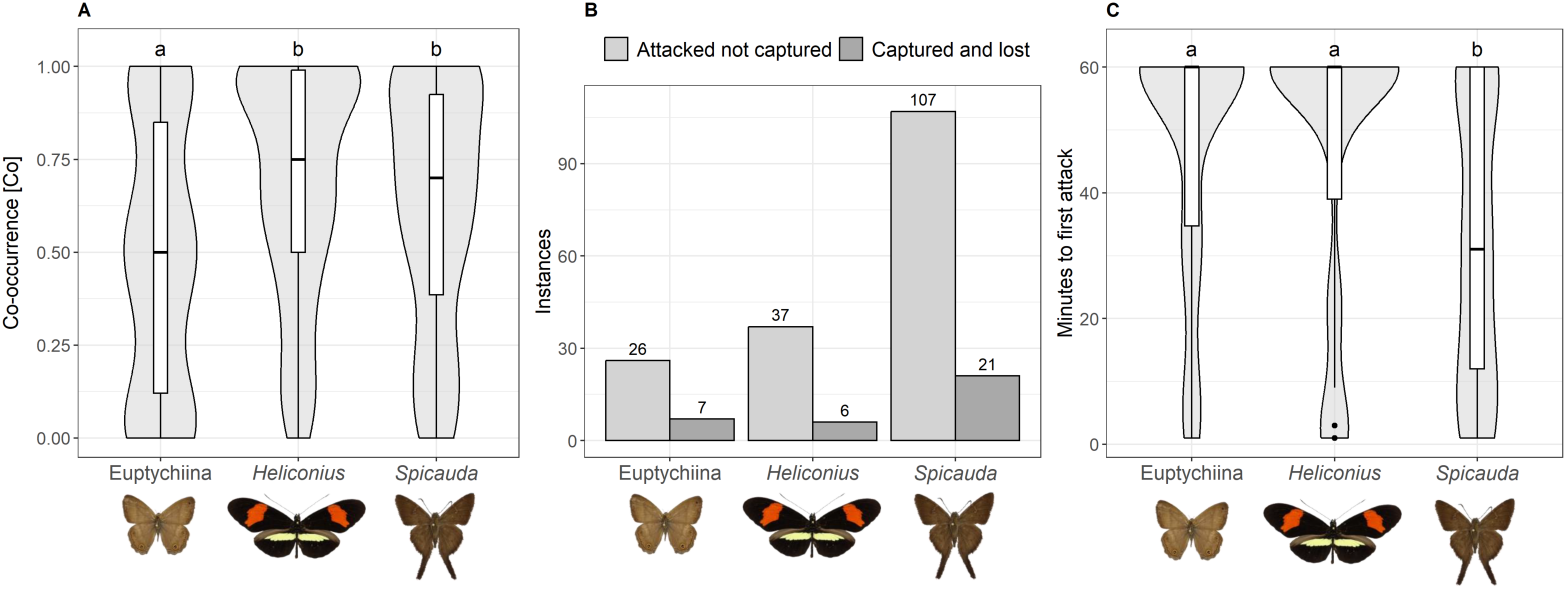
(A) Shared occupancy across high and low strata in the aviary for all three used butterfly types. Compact letters denote significant differences between groups (*p* < 0.05) based on the Wilcoxon signed‐rank test. (B) Sum of instances of attacking behavior without capture and capturing but subsequently losing the prey for each butterfly phenotype. (C) Duration until the first attack occurred for this butterfly type within the 60‐min experiment. Only for birds attacking at least one butterfly. Compact letters denote significant differences between butterfly types (Wilcoxon signed‐rank test). Butterfly pictures taken by Daniel Linke, photos in scale.

We used a nonparametric test to compare the encounter chance of bird and each butterfly (Co index, Figure 5A) between the three different prey types. Results showed that Euptychiines and *Heliconius* (pairwise Wilcoxon signed‐rank test, W = 5762; Bonferroni‐adjusted *p* = 1.2 × 10^−5^; Euptychiina's Co: avg. = 0.477, SD = 0.371 and *Heliconius*' Co: avg. = 0.641, SD = 0.344) as well as Euptychiines and *Spicauda* (pairwise Wilcoxon signed‐rank test, *W* = 6648.5; Bonferroni‐adjusted *p* = 0.00071; *Spicauda*'s Co: avg. = 0.600, SD = 0.351) differed significantly in their spatial co‐occurrence, whereas no difference was detected between *Spicauda* and *Heliconius* (pairwise Wilcoxon signed‐rank test, *W* = 9601; Bonferroni‐adjusted *p* = 0.283). This confirmed that while *Heliconius* and *Spicauda* co‐occurred in the aviary's upper part, Euptychiines preferred the ground, reducing their encounter probability.

*(2) Probability of being rejected upon identification*: The best‐selected model was “*Heliconius* different” (Akaike weight = 0.50; Figure 4, Table 1) with “all different” being the second‐best model (Akaike weight = 0.50, ΔAICc = 0.02). Both models suggested that most birds sight‐rejected *Heliconius*, followed by *Spicauda* and Euptychiines. A similar pattern was supported by both immatures and adults, with immatures exhibiting higher sight‐rejection rates toward all butterfly types. *Spicauda*, even though it co‐occurred at high rates with most birds in the aviaries, was sight‐rejected at a higher rate (the “all different” scenario) than Euptychiines.

*(3a) Targeting probability*: The “*Spicauda* different” was identified as the best model (Akaike weight = 0.62 Figure 4, Table 1), suggesting that birds more frequently attacked *Spicauda* than Euptychiines and *Heliconius*. This is in line with our observations of birds often attacking *Spicauda* repeatedly until they captured and subjugated it (Figure 5B); the ratio of the number of unsuccessful attacks to the sum of all birds attacking a prey is highest for *Spicauda* with 2.05, followed by *Heliconius* with 1.61 and Euptychiines with 0.92. The model “all different” had substantial support (Akaike weight = 0.25, ΔAICc = 1.77), suggesting that Euptychiines were attacked more frequently than *Heliconius* by birds that detected a butterfly prey. For adults, only the “all different” and “*Spicauda* different” models received substantial support (Akaike weights: 0.36 and 0.47, respectively), indicating *Spicauda* as the primary target. In contrast, immatures showed more even attack rates, with “all equal” being the best model (Akaike weight = 0.32) and “all different” was poorly supported (ΔAICc = 2.24, Akaike weight = 0.10), indicating less selective predatory responses.

*(3b) Capture avoidance*: “All equal” was the best model, suggesting that all butterflies showed similar likelihood of evading capture upon predator attack (Akaike weight = 0.37, Figure 4, Table 1). For immatures, additional models (“*Heliconius* different” and “Euptychiines different”) had ΔAICc between 1 and 2 (Akaike weights between 0.16 and 0.22), indicating varying capture efficiency. Conversely, for adult birds only “all equal” and “Euptychiina different” were selected (Akaike weights: 0.46 and 0.17, respectively), pointing to similar capture rates for *Heliconius* and *Spicauda*, with Euptychiines being the easiest to catch.

To supplement the likelihood‐based tests, we compared the times of first predator attack among butterfly types. The pairwise Wilcoxon signed‐rank tests suggested that the times to attack differed between *Spicauda* and *Heliconius* (*Spicauda* 32 ± 22.4 min; *Heliconius* 46.6 ± 22.3 min; *W* = 3627.5; Bonferroni‐adjusted *p* = 9.0 × 10^−5^) as well as between *Spicauda* and Euptychiines (Euptychiina 47.9 ± 19.6 min; *W* = 3728.5, Bonferroni‐adjusted *p* = 1.4 × 10^−5^) (Figure 5C), confirming that *Spicauda* was attacked significantly earlier compared to the other two prey types.

*(4) Probability to survive upon predator attack*: The “all equal” model was preferred (Akaike weight = 0.43), while most other models had ΔAICc >1.67 and Akaike weights <0.19. Only the model “all different” was rejected (ΔAICc = 3.79, Akaike weight = 0.06, Figure 4, Table 1). For immatures, “all equal” was preferred (Akaike weight = 0.40), while the models “*Heliconius* different,” “*Spicauda* different,” and “Euptychiina different” were not rejected (ΔAICc: 2.00, 1.77 and 1.18; Akaike weights: 0.15, 0.16 and 0.22, respectively), suggesting that Euptychiines were more likely to be subjugated once a predator attacked it compared to *Spicauda* or *Heliconius*. This pattern was absent for adults, with the “all equal” scenario being the only model with ΔAICc <2 (Akaike weight: 0.47).

Results of log‐likelihood tests between habitats (forest vs. urban) and seasons (dry vs. wet) can be found in Appendix S1: Section S7: Table S4. Stacked bar graphs between seasons and habitats can be found in Appendix S1: Section S8: Figure S8.

## DISCUSSION

Understanding how prey defenses function across the different stages of predator encounters is fundamental to explaining species persistence, interactions, and evolution within communities. In this study, we tested four predictions derived from the predation sequence, focusing on the fitness benefits and functional roles of antipredator defenses during the stages of encounter, identification, attack, and subjugation. We studied tropical birds that include insects in their diets and live butterflies displaying antipredator defenses (crypsis, aposematism, and evasiveness) thought to function best at different stages of the predation sequence (Kikuchi et al., 2023). In controlled aviary settings, studying the local community of wild insectivorous birds, we confirm that crypsis reduces the likelihood of encounter and detection, aposematism deters attacks, and evasive flight reduces the probability of subjugation after attacks. However, we also provide evidence that bird age and, to some degree, diet are important predictors of defense effectiveness. Aposematism was less effective against immature birds, while evasive ability was more successful in avoiding attacks from immatures. Finally, we quantify similar survival rates across all predation stages, regardless of antipredator defense. The higher number of insectivorous birds and larger proportion of immatures in the mature secondary forest, compared to the urban environment, were key drivers of the similar mortality rates across butterfly types.

### Defense efficiency along the predation sequence

*(1) Probability to remain undetected*: Cryptic species often remain motionless for prolonged periods of time, are hard to spot (Ruxton et al., 2018; Seymoure et al., 2018; Stevens & Merilaita, 2009), and forage close to the ground (Burd, 1994). Euptychiines are characterized by dull wing colorations, which, indeed, resulted in them being the least likely prey phenotype to be encountered by birds in our study. Conversely, while *Spicauda* also has a uniform brown appearance and might be considered cryptic, their conspicuous flight behavior often renders them in spatial proximity to most avian predators, thus possibly indicating a relaxed selection for crypsis and a reliance on their evasive flight abilities or hindwing tails to evade predation upon attack.

We detected a strong preference of fully or partly insectivorous birds toward the upper layer of the aviary (200–400 cm). Although the detection rates of prey varied in experiments carried out in the forest and urban environments, this might be due to differences in species‐specific height preferences of birds or the different abundances of immatures relative to adults in each habitat. Due to limited overlap in species and age distribution between study locations, we cannot determine why prey detection rates shifted across habitats (Appendix S1: Section S7: Table S4). Future studies should survey birds over longer periods and in various locations and across habitats, to compare differences in foraging strategies among species and life stages. Including other skilled insect‐hunting birds in behavioral experiments, such as Jacamars (Galbulidae) (Chai, 1986; Langham, 2006; Pinheiro & Campos, 2019), which were absent at our study locations, would provide further insights on antipredator defense evolution using a full spectrum of predator types.

Altogether, our results emphasize that immature birds, possibly due to their naivety, are less prone to detect cryptic prey compared to more experienced adults (Mappes et al., 2014). Overall, the effectiveness of crypsis in evading detection is evident, particularly in diverse environments where immature birds dominate, as in our study location in the mature secondary forest.

*(2) Probability of being rejected upon identification*: Our results show that regardless of bird diet and species, predators often sight rejected *Heliconius* (i.e., there was no attack behavior with predator and prey being closer than ~10 cm). This suggests that wild birds recognized the aposematic local patterns of unprofitable prey (e.g., Dell'aglio et al., 2016; Finkbeiner et al., 2014; Merrill et al., 2012). Nevertheless, immature birds, presumably being more naïve, exhibited a less discriminating predation pattern, aligning with the findings of Mappes et al. (2014), where aposematism is more favorable during seasons with low numbers of naïve birds. In tropical habitats, naïve birds are present throughout the year (Stouffer et al., 2013). However, this also creates an almost continuous selective pressure on aposematic signals during the year and may be linked to the evolution of diverse warning cues in the tropics.

In laboratory experiments, birds can learn phenotypic cues associated with escape ability and change their predation behavior to avoid such evasive prey (Linke et al., 2022; Loeffler‐Henry & Sherratt, 2024; Páez et al., 2021). Additionally, phenotypic cues commonly found in evasive neotropical butterflies, such as dorsal iridescence, forewing bands, and hindwing tails, might advertise difficulty of capture (Janzen et al., 2009; Linke et al., 2022; Pinheiro & Freitas, 2014). While testing avoidance due to evasive capabilities has seldom been tested with wild predators (Guerra et al., 2024), our results hint at some avoidance by immature birds. Nonetheless, attack avoidance of evasive prey might be higher in species less adept at catching flying insects (such as Cardinalidae or Thraupidae) but due to our low sample size and unequal abundances of immatures and adults across habitats, this speculation remains to be corroborated.

*(3) Targeting probability and capture avoidance*: Although there are slight variations in capture rates between immature and adult birds, these differences are relatively minor. This suggests that all three prey phenotypes were equally susceptible to be captured when attacked, regardless of the birds' experience. This equal likelihood of capture could also be explained by the restricted space in our aviaries available for prey to escape capture. However, we found that adults were slightly better than immatures at catching the evasive prey *Spicauda*, as expected under the age‐specific foraging proficiency concept (Wunderle, 1991).

Attack rates, on the other hand, diverge drastically between adult and immature birds. Adults preferred attacking *Spicauda* and refrained from attacking *Heliconius*, while immatures had similar attack rates across all three prey phenotypes. Learning and memorizing aposematic cues of unprofitable prey is well‐documented in behavioral experiments in the laboratory (Gibson, 1974, 1980; Hansen et al., 2010; Linke et al., 2022; Páez et al., 2021; Skelhorn & Rowe, 2006; Zvereva & Kozlov, 2021), including tests on birds of different age classes (Langham, 2006; Veselý et al., 2017). Because the precise age determination of birds is impractical in the wild, immature individuals in our study may have encompassed a spectrum ranging from recently fledged to residents of up to 2 years. However, predatorial behaviors in wild birds have rarely been assessed using age groups (Guerra et al., 2024) or were limited in sample size (Pinheiro & Campos, 2019). Thus, to our knowledge, age‐related variation in prey attack and how it shapes the evolution and co‐existence of multiple defenses have received little empirical support.

Contrary to expectations, *Spicauda* was just as likely to be caught by insectivorous birds as the other prey types, despite its presumed evasive abilities. Nevertheless, the total instances of failed attacks are considerably higher for *Spicauda* compared to *Heliconius* or Euptychiines, pointing toward a bigger struggle for the birds to catch and subjugate *Spicauda* (Figure 5C). The closed experimental space and food deprivation of birds prior to the experiment might have increased the mortality of *Spicauda* compared to natural conditions; in nature, unsuccessful attacks would be less likely to be followed by more attempts. However, high attack rates on evasive species might be explained by high profitability, especially for agile predators. Evasive species with high flying speed typically have high wing loading (see review: Le Roy et al., 2019; expected flight speed of skipper species including *Spicauda*: Linke et al., 2024), which can be driven by increased body mass. Thus, such evasive species might still be profitable to chase, though not for less agile or naïve predators.

*(4) Probability to survive upon predator attack*: Mortality rates were relatively even across prey phenotypes and bird age groups. While log‐likelihood tests detected some differences among immatures, they were completely absent among adults, hinting at *Heliconius* and *Spicauda* being less likely to be killed when attacked by immature birds. *Heliconius* were rejected after attacks due to chemical defenses, while *Spicauda* were harder to catch and subjugate and were less likely to be killed by less skilled predators. This is at least partly in agreement with our initial prediction, as species with high evasive capabilities or chemical defenses are less likely to be killed by predators, at least among immature birds. Additionally, it points toward predation pressure being more equally distributed in more diverse habitats, which has been supported for aquatic habitats but less so for terrestrial, tropical ones (Chang & Todd, 2023).

Our findings demonstrate that multiple antipredator strategies—crypsis, aposematism, and evasive behavior—can be equally effective for survival in tropical forest ecosystems, albeit in different ways and at different stages of the predation process. The relatively similar mortality rate across prey types suggests that effective predation pressure is relatively even, allowing diverse prey strategies to diversify and coexist. This pattern reflects the complexity of tropical food webs, where the high diversity of both predators and prey likely promotes the persistence of multiple defensive adaptations. The role of predator age further underscores how variation in predator experience and behavior shapes the frequency of prey defenses. Altogether, these results highlight how predation contributes to the ecological and evolutionary processes that sustain the large diversity of antipredator defenses found in the tropics.

## AUTHOR CONTRIBUTIONS

Daniel Linke: study planning, field investigation, data curation and evaluation, video processing, statistical analysis, writing original draft, and review and editing; Jacqueline Hernandez Mejia: field investigation and review and editing; Valery N. P. Eche Navarro: field investigation and review and editing; Prapti Gohil: video processing, writing, and editing; César Ramírez García: field investigation; Letty Salinas: study planning, supervision, and review and editing; Marianne Elias: study planning, statistical analysis, and review and editing; Pável Matos‐Maraví: study planning, field investigation, funding acquisition, and review and editing.

## FUNDING INFORMATION

Funding was provided by the Junior GAČR grant (GJ20‐18566Y), the PPLZ program of the Czech Academy of Sciences (fellowship grant L20096195), and Grantová Agentura Jihočeské Univerzity n.014/2022/P. Logistic support in Peru was supported by 421 Fundación San Marcos, Letty Salinas was partially funded by Universidad Nacional Mayor de San Marcos RR 05557‐R‐22 (Project B22100321).

## CONFLICT OF INTEREST STATEMENT

The authors declare no conflicts of interest.

## Supporting information


Appendix S1:

## Data Availability

Data (Linke, 2025) are available in Zenodo at https://doi.org/10.5281/zenodo.17054629.

## References

[ecy70248-bib-0001] Arias, M. , J. Mappes , M. Théry , and V. Llaurens . 2016. “Inter‐Species Variation in Unpalatability Does Not Explain Polymorphism in a Mimetic Species.” Evolutionary Ecology 30(3): 419–433. 10.1007/s10682-015-9815-2.

[ecy70248-bib-0002] Bates, D. , M. Mächler , B. Bolker , and S. Walker . 2015. “Fitting Linear Mixed‐Effects Models Using lme4.” Journal of Statistical Software 67: 1–48. 10.18637/jss.v067.i01.

[ecy70248-bib-0003] Burd, M. 1994. “Butterfly Wing Colour Patterns and Flying Heights in the Seasonally Wet Forest of Barro Colorado Island, Panama.” Journal of Tropical Ecology 10(4): 601–610. 10.1017/S0266467400008270.

[ecy70248-bib-0004] Burnham, K. P. , and D. R. Anderson . 2002. Model Selection and Inference: A Practical Information‐Theoretic Approach. New York, NY: Springer Science & Business Media.

[ecy70248-bib-0005] Chai, P. 1986. “Field Observations and Feeding Experiments on the Responses of Rufous‐Tailed Jacamars (Galbula Ruficauda) to Free‐Flying Butterflies in a Tropical Rainforest.” Biological Journal of the Linnean Society 29(3): 161–189. 10.1111/j.1095-8312.1986.tb01772.x.

[ecy70248-bib-0006] Chang, C. , and P. A. Todd . 2023. “Reduced Predation Pressure as a Potential Driver of Prey Diversity and Abundance in Complex Habitats.” npj Biodiversity 2(1): 1–5. 10.1038/s44185-022-00007-x. 39242650 PMC11332019

[ecy70248-bib-0007] Chotard, A. , J. Ledamoisel , T. Decamps , A. Herrel , A. S. Chaine , V. Llaurens , and V. Debat . 2022. “Evidence of Attack Deflection Suggests Adaptive Evolution of Wing Tails in Butterflies.” Proceedings of the Royal Society B: Biological Sciences 289(1975): 20220562. 10.1098/rspb.2022.0562. PMC9130794 35611535

[ecy70248-bib-0008] Chouteau, M. , M. Arias , and M. Joron . 2016. “Warning Signals Are under Positive Frequency‐Dependent Selection in Nature.” Proceedings of the National Academy of Sciences 113(8): 2164–2169. 10.1073/pnas.1519216113. PMC4776528 26858416

[ecy70248-bib-0009] Chouteau, M. , J. Dezeure , T. N. Sherratt , V. Llaurens , and M. Joron . 2019. “Similar Predator Aversion for Natural Prey with Diverse Toxicity Levels.” Animal Behaviour 153: 49–59. 10.1016/j.anbehav.2019.04.017.

[ecy70248-bib-0010] Dell'aglio, D. D. , M. Stevens , and C. D. Jiggins . 2016. “Avoidance of an Aposematically Coloured Butterfly by Wild Birds in a Tropical Forest.” Ecological Entomology 41(5): 627–632. 10.1111/een.12335. 27708481 PMC5026159

[ecy70248-bib-0011] Egg, A. B. , T. A. Parker , J. P. O'Neill , D. F. Lane , D. F. Stotz , and T. S. Schulenberg . 2010. Birds of Peru: Revised and Updated Edition. Princeton, NJ: Princeton University Press. https://muse.jhu.edu/pub/267/monograph/book/30246.

[ecy70248-bib-0012] Endler, J. A. 1991. “Interactions Between Predators and Prey.” In Behavioural Scology: An Evolutionary Approach, 3rd ed., edited by J. R. Krebs and N. B. Davies , 169–196. Oxford: Blackwell.

[ecy70248-bib-0013] Feltmate, B. W. , and D. D. Williams . 1989. “A Test of Crypsis and Predator Avoidance in the Stonefly Paragnetina Media (Plecoptera: Perlidae).” Animal Behaviour 37: 992–999. 10.1016/0003-3472(89)90143-7.

[ecy70248-bib-0014] Finkbeiner, S. D. , A. D. Briscoe , and R. D. Reed . 2014. “Warning Signals Are Seductive: Relative Contributions of Color and Pattern to Predator Avoidance and Mate Attraction in Heliconius Butterflies.” Evolution 68(12): 3410–3420. 10.1111/evo.12524. 25200939

[ecy70248-bib-0015] Gibson, D. O. 1974. “Batesian Mimicry without Distastefulness?” Nature 250(5461): 77–79. 10.1038/250077a0. 4841589

[ecy70248-bib-0016] Gibson, D. O. 1980. “The Role of Escape in Mimicry and Polymorphism: I. The Response of Captive Birds to Artificial Prey.” Biological Journal of the Linnean Society 14(2): 201–214. 10.1111/j.1095-8312.1980.tb00105.x.

[ecy70248-bib-0017] Guerra, T. J. , R. F. Braga , F. Camarota , F. S. Neves , and G. W. Fernandes . 2024. “Avian Predators Avoid Attacking Fly‐Mimicking Beetles: A Field Experiment on Evasive Mimicry Using Artificial Prey.” The American Naturalist 204(1): 96–104. 10.1086/730263. 38857342

[ecy70248-bib-0018] Hansen, B. T. , Ø. H. Holen , and J. Mappes . 2010. “Predators Use Environmental Cues to Discriminate between Prey.” Behavioral Ecology and Sociobiology 64(12): 1991–1997. 10.1007/s00265-010-1010-4.

[ecy70248-bib-0051] Hernandez Mejia, J. , V. N. P. Eche Navarro , D. Linke , C. Ramírez García , L. Salinas Sánchez , and P. Matos‐Maraví . 2025. Birds of Urahuasha Protected Area, Vol. 1737, 24. Chicago, Illinois, USA: Field Guides, Field Museum.

[ecy70248-bib-0019] Janzen, D. H. , W. Hallwachs , P. Blandin , J. M. Burns , J.‐M. Cadiou , I. Chacon , T. Dapkey , et al. 2009. “Integration of DNA Barcoding into an Ongoing Inventory of Complex Tropical Biodiversity.” Molecular Ecology Resources 9(s1): 1–26. 10.1111/j.1755-0998.2009.02628.x. 21564960

[ecy70248-bib-0053] Kikuchi, D. W. , W. L. Allen , K. Arbuckle , T. G. Aubier , E. S. Briolat , E. R. Burdfield‐Steel , K. L. Cheney , et al. 2023. “The Evolution and Ecology of Multiple Antipredator Defences.” Journal of Evolutionary Biology 36: 975–991. 10.1111/jeb.14192. 37363877

[ecy70248-bib-0020] Langham, G. M. 2006. “Rufous‐Tailed Jacamars and Aposematic Butterflies: Do Older Birds Attack Novel Prey?” Behavioral Ecology 17(2): 285–290. 10.1093/beheco/arj027.

[ecy70248-bib-0021] Le Roy, C. , V. Debat , and V. Llaurens . 2019. “Adaptive Evolution of Butterfly Wing Shape: From Morphology to Behaviour.” Biological Reviews 94(4): 1261–1281. 10.1111/brv.12500. 30793489

[ecy70248-bib-0022] Lenth, R. 2025. “emmeans: Estimated Marginal Means, aka Least‐Squares Means. (Version 1.11.1‐00001) [Computer software].” https://rvlenth.github.io/emmeans/

[ecy70248-bib-0023] Linke, D. 2025. “Quantifying the Success of Prey Crypsis, Aposematism and Evasiveness in Avoiding Predators Attack.” Zenodo. 10.5281/zenodo.17054629 41260231

[ecy70248-bib-0024] Linke, D. , M. Elias , I. Klečková , J. Mappes , and P. Matos‐Maraví . 2022. “Shape of Evasive Prey Can Be an Important Cue that Triggers Learning in Avian Predators.” Frontiers in Ecology and Evolution 10: 910695. 10.3389/fevo.2022.910695.

[ecy70248-bib-0025] Linke, D. , J. Hernandez Mejia , V. N. P. Eche Navarro , L. Salinas Sánchez , P. de Gusmão Ribeiro , M. Elias , and P. Matos‐Maraví . 2024. “Reduced Palatability, Fast Flight, and Tails: Decoding the Defence Arsenal of Eudaminae Skipper Butterflies in a Neotropical Locality.” Journal of Evolutionary Biology 37: voae091. 10.1093/jeb/voae091. 39044333

[ecy70248-bib-0026] Loeffler‐Henry, K. , and T. N. Sherratt . 2024. “Selection for Evasive Mimicry Imposed by an Arthropod Predator.” Biology Letters 20(1): 20230461. 10.1098/rsbl.2023.0461. 38166416 PMC10762431

[ecy70248-bib-0027] Mallet, J. , and L. Gilbert . 1995. “Why Are there So Many Mimicry Rings? Correlations between Habitat, Behaviour and Mimicry in Heliconius Butterflies.” Biological Journal of the Linnean Society 55(2): 159–180. 10.1111/j.1095-8312.1995.tb01057.x.

[ecy70248-bib-0028] Mappes, J. , H. Kokko , K. Ojala , and L. Lindström . 2014. “Seasonal Changes in Predator Community Switch the Direction of Selection for Prey Defences.” Nature Communications 5(1): Article 1. 10.1038/ncomms6016. PMC4199109 25247589

[ecy70248-bib-0029] Mérot, C. , B. Frérot , E. Leppik , and M. Joron . 2015. “Beyond Magic Traits: Multimodal Mating Cues in Heliconius Butterflies.” Evolution 69(11): 2891–2904. 10.1111/evo.12789. 26513426

[ecy70248-bib-0030] Merrill, R. M. , R. W. R. Wallbank , V. Bull , P. C. A. Salazar , J. Mallet , M. Stevens , and C. D. Jiggins . 2012. “Disruptive Ecological Selection on a Mating Cue.” Proceedings of the Royal Society B: Biological Sciences 279(1749): 4907–4913. 10.1098/rspb.2012.1968. PMC3497240 23075843

[ecy70248-bib-0031] Molleman, F. , J. Javoiš , R. B. Davis , M. R. L. Whitaker , T. Tammaru , A. Prinzing , E. Õunap , et al. 2020. “Quantifying the Effects of Species Traits on Predation Risk in Nature: A Comparative Study of Butterfly Wing Damage.” Journal of Animal Ecology 89(3): 716–729. 10.1111/1365-2656.13139. 31693172

[ecy70248-bib-0032] Navarro, V. , J. Hernández Mejía , D. Linke , L. Salinas , and P. Matos‐Maraví . 2023. “*Birds of Tarapoto (San Martín, Peru)* (1640; p. 12). Field Guides, Field Museum.” 10.13140/RG.2.2.14541.20964

[ecy70248-bib-0033] Páez, E. , J. K. Valkonen , K. R. Willmott , P. Matos‐Maraví , M. Elias , and J. Mappes . 2021. “Hard to Catch: Experimental Evidence Supports Evasive Mimicry.” Proceedings of the Royal Society B: Biological Sciences 288(1946): 20203052. 10.1098/rspb.2020.3052. PMC7944090 33715434

[ecy70248-bib-0034] Pembury Smith, M. Q. R. , and G. D. Ruxton . 2021. “Size‐Dependent Predation Risk in Cryptic Prey.” Journal of Ethology 39(2): 191–198. 10.1007/s10164-021-00691-5.

[ecy70248-bib-0035] Pinheiro, C. E. G. , and V. C. Campos . 2019. “The Responses of Wild Jacamars (Galbula Ruficauda, Galbulidae) to Aposematic, Aposematic and Cryptic, and Cryptic Butterflies in Central Brazil.” Ecological Entomology 44(4): 441–450. 10.1111/een.12723.

[ecy70248-bib-0036] Pinheiro, C. E. G. , and R. Cintra . 2017. “Butterfly Predators in the Neotropics: Which Birds Are Involved?” Journal of the Lepidopterists' Society 71: 109–114. 10.18473/lepi.71i2.a5.

[ecy70248-bib-0037] Pinheiro, C. E. G. , and A. V. L. Freitas . 2014. “Some Possible Cases of Escape Mimicry in Neotropical Butterflies.” Neotropical Entomology 43(5): 393–398. 10.1007/s13744-014-0240-y. 27193948

[ecy70248-bib-0052] R Core Team . 2023. R: A Language and Environment for Statistical Computing. Vienna: R Foundation for Statistical Computing. https://www.R-project.org/.

[ecy70248-bib-0038] Ruxton, G. D. , W. L. Allen , T. N. Sherratt , and M. P. Speed . 2018. Avoiding Attack: The Evolutionary Ecology of Crypsis, Aposematism, and Mimicry, 2nd ed. Oxford: Oxford University Press. 10.1093/oso/9780199688678.001.0001.

[ecy70248-bib-0039] Ruxton, G. D. , M. Speed , and T. N. Sherratt . 2004. “Evasive Mimicry: When (if Ever) Could Mimicry Based on Difficulty of Capture Evolve?” Proceedings. Biological sciences 271(1553): 2135–2142. 10.1098/rspb.2004.2816. 15475333 PMC1691841

[ecy70248-bib-0040] Seymoure, B. M. , A. Raymundo , K. J. McGraw , W. Owen McMillan , and R. L. Rutowski . 2018. “Environment‐Dependent Attack Rates of Cryptic and Aposematic Butterflies.” Current Zoology 64(5): 663–669. 10.1093/cz/zox062. 30323845 PMC6178784

[ecy70248-bib-0041] Skelhorn, J. , and C. Rowe . 2006. “Prey Palatability Influences Predator Learning and Memory.” Animal Behaviour 71(5): 1111–1118. 10.1016/j.anbehav.2005.08.011.

[ecy70248-bib-0042] Srygley, R. B. , and P. Chai . 1990. “Predation and the Elevation of Thoracic Temperature in Brightly Colored Neotropical Butterflies.” The American Naturalist 135(6): 766–787.

[ecy70248-bib-0043] Stevens, M. , and S. Merilaita . 2009. “Animal Camouflage: Current Issues and New Perspectives.” Philosophical Transactions of the Royal Society B: Biological Sciences 364(1516): 423–427. 10.1098/rstb.2008.0217. PMC2674078 18990674

[ecy70248-bib-0044] Stouffer, P. , E. Johnson , and R. Bierregaard . 2013. “Breeding Seasonality in Central Amazonian Rainforest Birds.” The Auk 130: 529–540. 10.1525/auk.2013.12179.

[ecy70248-bib-0045] Vallin, A. , S. Jakobsson , J. Lind , and C. Wiklund . 2006. “Crypsis Versus Intimidation—Anti‐Predation Defence in Three Closely Related Butterflies.” Behavioral Ecology and Sociobiology 59(3): 455–459. 10.1007/s00265-005-0069-9.

[ecy70248-bib-0046] Veselý, P. , B. Ernestová , O. Nedvěd , and R. Fuchs . 2017. “Do Predator Energy Demands or Previous Exposure Influence Protection by Aposematic Coloration of Prey?” Current Zoology 63(3): 259–267. 10.1093/cz/zow057. 29491984 PMC5804175

[ecy70248-bib-0047] Wickham, H. 2016. Ggplot2. Cham: Springer International Publishing. 10.1007/978-3-319-24277-4.

[ecy70248-bib-0048] Willmott, K. R. , J. C. Robinson Willmott , M. Elias , and C. D. Jiggins . 2017. “Maintaining Mimicry Diversity: Optimal Warning Colour Patterns Differ among Microhabitats in Amazonian Clearwing Butterflies.” Proceedings. Biological sciences 284(1855): 20170744. 10.1098/rspb.2017.0744. 28539522 PMC5454276

[ecy70248-bib-0049] Wunderle, J. 1991. “Age‐Specific Foraging Proficiency in Birds.” Current Ornithology 8: 273–324.

[ecy70248-bib-0050] Zvereva, E. L. , and M. V. Kozlov . 2021. “Seasonal Variations in Bird Selection Pressure on Prey Colouration.” Oecologia 196(4): 1017–1026. 10.1007/s00442-021-04994-9. 34322748 PMC8367932

