# Supplementary material for: Quantifying the success of prey crypsis, aposematism, and evasiveness in avoiding predator attack
Source: Ecology. 2025 Nov 19;106(11):e70248. doi: 10.1002/ecy.70248 (PMC12629670; doi:10.1002/ecy.70248)
Supplement: Supplementary file 1 — Appendix S1: [file ECY-106-e70248-s001.pdf]

Appendix S1

Quantifying the success of prey crypsis, aposematism, and evasiveness in avoiding predator attack

Daniel Linke, Jacqueline Hernandez Mejia, Valery N. P. Eche Navarro, Prapti Gohil, César Ramírez García, Letty Salinas, Marianne Elias, Pável Matos-Maraví

Ecology

Section S1: Coordinates of all nets used to trap low flying birds

Table S1: Nets used to trap birds from the urban environment during the rainy season from October 2021 to February 2022.

Name of the net	Longitude	Latitude	Altitude [masl]	Site description
casa	-76.35609576	-6.478592891	380	Forest edge
1 casa	-76.35618622	-6.478610737	381	Forest edge
2 casa	-76.35622227	-6.478565422	383	Forest edge
3-4 casa	-76.35617694	-6.478520325	382	Forest edge
5 casa	-76.35615893	-6.478547504	382	Forest edge
6 casa	-76.35613420	-6.479442893	370	Forest edge
7 casa	-76.35607095	-6.479461150	368	Forest edge
8 casa	-76.35626054	-6.479352119	373	Forest edge
9-10 casa	-76.35633302	-6.479406187	374	Forest edge
1-2 shilcayo	-76.35547340	-6.475772927	378	Open grassland
3-4-5 shilcayo	-76.35505776	-6.475873517	360	Open grassland
1 suchiche	-76.35799049	-6.483851238	351	Relict forest
2 suchiche	-76.35799063	-6.483905499	351	Relict forest

Table S2: Nets used to trap birds from the forest environment during the rainy season from October 2021 to February 2022 and during the dry season from June to September 2022.

Name of the net	Longitude	Latitude	Altitude [masl]	Site description
1 rio	-76.33184732	-6.465299459	674	Closed secondary forest
2 rio	-76.33191080	-6.465371643	673	Closed secondary forest
piña chacra	-76.33513614	-6.460986010	714	Forest edge
1-2 caseta	-76.33624607	-6.463605785	724	Low density secondary forest
3 caseta	-76.33614585	-6.463316648	723	Closed secondary forest
4 caseta	-76.33618128	-6.463036199	724	Closed secondary forest
5 caseta	-76.33607416	-6.463561018	721	Closed secondary forest
6-7 caseta	-76.33631840	-6.463605595	725	Low density secondary forest
tambo	-76.33347959	-6.460230678	751	Clearing of secondary forest
1 chacra	-76.33379675	-6.460501159	740	Low density secondary forest
2 chacra	-76.33394141	-6.460500780	737	Low density secondary forest
3 chacra	-76.33413028	-6.460120447	732	Low density secondary forest
4 chacra	-76.33401424	-6.460690508	734	Low density secondary forest
5 chacra	-76.33409565	-6.460708382	732	Low density secondary forest
6 chacra	-76.33423194	-6.460961250	730	Low density secondary forest
7-8 chacra	-76.33383002	-6.459397734	739	Clearing of secondary forest
9-10 chacra	-76.33376704	-6.459515468	741	Clearing of secondary forest
11-12 chacra	-76.33361708	-6.460944774	740	Low density secondary forest
13 chacra	-76.33370711	-6.460799838	740	Low density secondary forest

Section S2: Photos of the aviaries in the urban and forested localities.

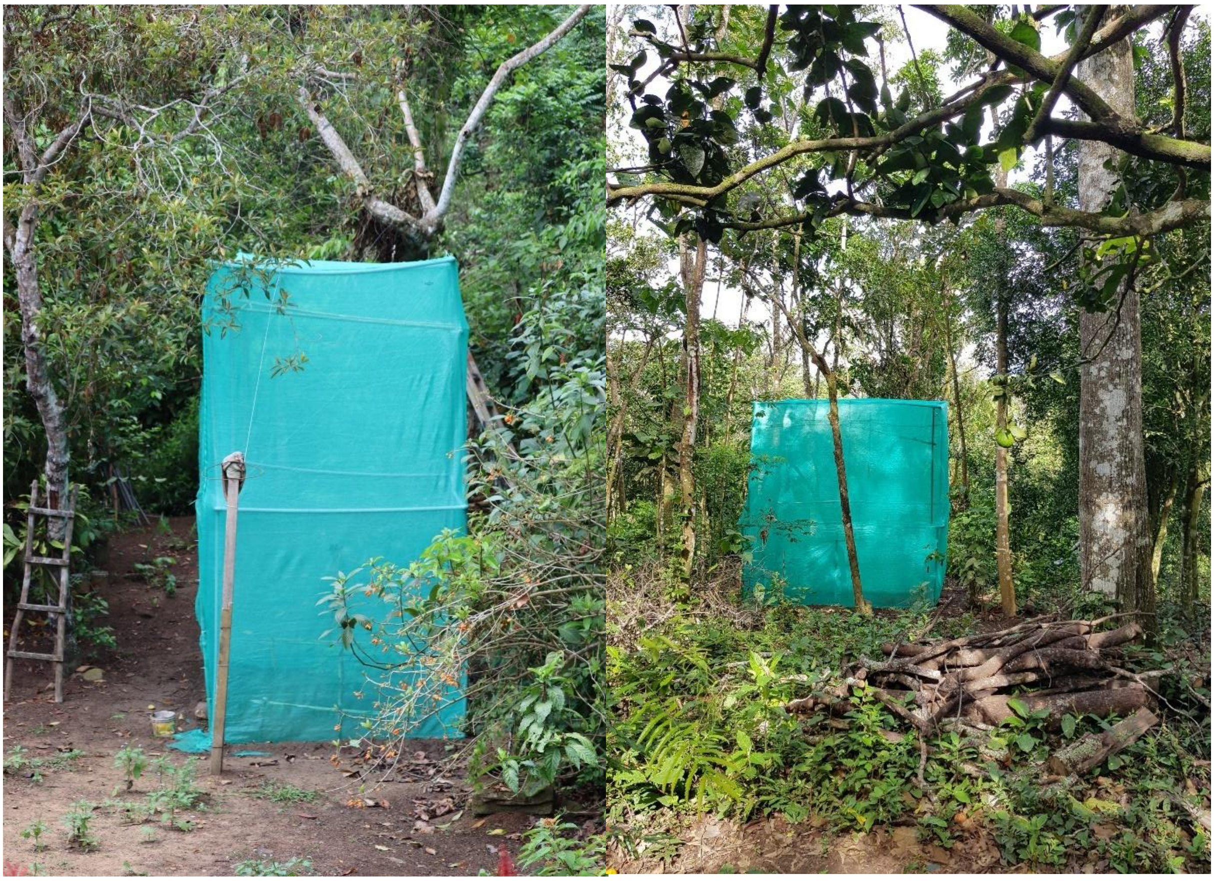

Figure S1: Aviary at the urban (left) and forested (right) study sites. Outer dimensions approximately: height = 4m, depth = 2m and length = 4m. Tubing in the middle marked the 200 cm height used to separate the higher and lower stratum. Bamboo perch for bird to rest was installed approximately 50cm below the mesh ceiling. Photos by D. Linke.

Section S3: List of bird species used for valid experiments depending on habitat, season and age.

Table S3: Breakdown of bird species used in successful experiments according to bird family and experimental season / habitat. Number in brackets represents the number of adult and immature individuals (Syntax = Adult-Immature). Diets classes were condensed for GLMM models to insectivorous and other (comprising all other diet classes).

	Urban-wet	Forest-wet	Forest-dry	Diet
**Bucconidae** 1 (1-0)				
Monasa morphoeus			1 (1-0)	insectivorous
**Cardinalidae** 8 (1-7)				
Chlorothraupis frenata			1 (0-1)	frugivore
Habia rubica		5 (0-5)	1 (1-0)	frugivore/invertebrate
Piranga olivacea	1 (0-1)			frugivore/invertebrate
**Cotingidae** 2 (0-2)				
Lipaugus vociferans		2 (0-2)		frugivore/invertebrate
**Furnariidae** 20 (2-18)				
Dendrocincla fuliginosa		1 (0-1)	3 (0-3)	insects/omnivore
Dendroma erythorptera		1 (0-1)		omnivore
Furnarius leucopus	1 (1-0)			insectivorous
Glyphorynchus spirurus			3 (0-3)	insectivorous
Sittasomus griseicapillus			1 (0-1)	frugivore/invertebrate
Xenops minutus			5 (0-5)	insectivorous
Xiphorhynchus elegans		2 (1-1)	3 (0-3)	insects/omnivore
**Icteridae** 2 (2-0)				
Cacicus cela	2 (2-0)			insects/omnivore
**Pipromorphidae** 50 (8-42)				
Leptopogon amaurocephalus		9 (4-5)	11 (2-9)	insectivorous
Mionectes oleagineus		5 (2-3)	24 (0-24)	frugivore/invertebrate
Mionectes olivaceus			1 (0-1)	frugivore/invertebrate
**Thamnophilidae** 24 (12-12)				
Hypocnemis peruviana			1 (0-1)	insectivorous
Myrmelastes leucostigma			1 (1-0)	insectivorous
Myrmoborus myotherinus		2 (0-2)	1 (1-0)	insectivorous
Myrmotherula axillaris			6 (2-4)	insectivorous
Pithys albifrons			2 (0-2)	insectivorous
Sciaphylax hemimelaena		2 (2-0)	2 (2-0)	frugivore/invertebrate
Thamnophilus schistaceus		1 (1-0)	2 (0-2)	insectivorous
Thamnophilus unicolor		1 (1-0)		frugivore
Willisornis poecilinotus		1 (1-0)	2 (1-1)	insectivorous
**Thraupidae** 27 (25-2)				
Saltator maximus		1 (1-0)	2 (1-1)	frugivore/invertebrate
Thraupis episcopus	18 (17-1)			frugivore/invertebrate
Thraupis palmarum	6 (6-0)			frugivore/invertebrate
**Tityridae** 2 (2-0)				
Myiobius barbatus		1 (1-0)		insectivorous

Pachyramphus polychopterus		1 (1-0)		insectivorous
**Troglodytidae** 1 (0-1)				
Microcerculus marginatus			1 (0-1)	insectivorous
**Turdidae** 32 (0-32)				
Catharus ustulatus	3 (0-3)	29 (0-29)		frugivore/invertebrate
**Tyrannidae** 38 (21-17)				
Attila spadiceus		3 (3-0)		omnivore
Elaenia parvirostris	1 (1-0)			insects/omnivore
Myiodynastes maculatus			2 (0-2)	insects/omnivore
Myiozetetes similis	3 (3-0)			frugivore/invertebrate
Pitangus sulphuratus	23 (12-11)			omnivore
Rhynchocyclus olivaceus			5 (1-4)	insectivorous
Tyrannus melancholicus	1 (1-0)			insectivorous
**Vireonidae** 9 (3-6)				
Vireo flavoviridis	3 (0-3)	1 (0-1)		frugivore/invertebrate
Vireo olivaceus	3 (1-2)	2 (2-0)		frugivore/invertebrate

Section S4: Overview of bird-butterfly-interactions along the predation sequence (not encountered, sight-rejected, attacked, caught, subjugated) depending on bird diet (Insectivorous vs. Other) and bird family

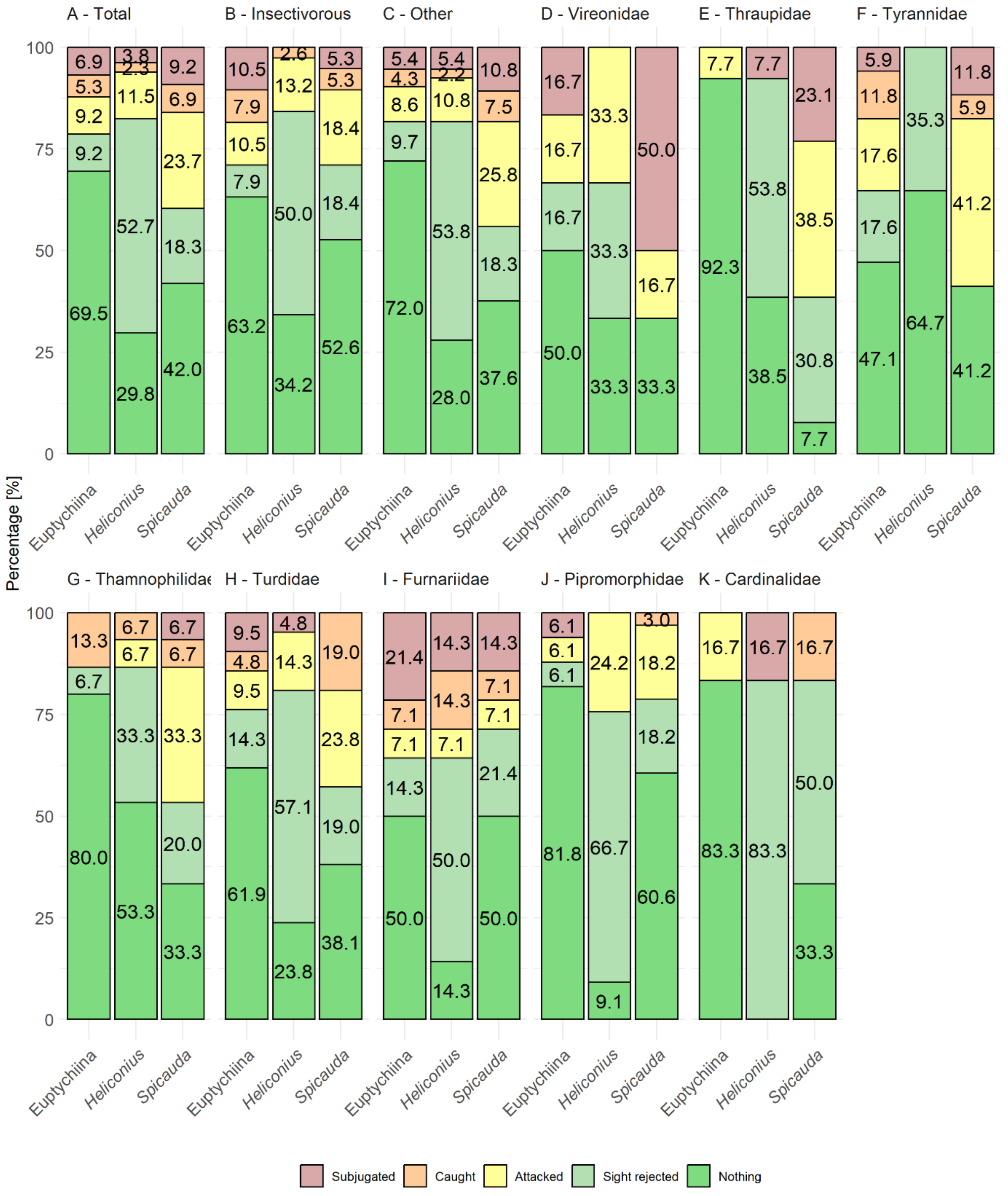

Figure S2: Overview of the highest predator-prey-interaction (%) observed between prey phenotypes depending on the diet and bird family. Results for bird families are sorted from the highest attacking behaviour towards Spicauda to lowest. Only including families with 5 or more

birds and birds which interacted with at least one prey phenotype, total and diet (insectivorous and other) includes bird families with less than 5 tested individuals.

Section S5: Logistic regression models

The formulas "*sight rejection / attacking ~ butterfly_type + state + diet_reduced + (1|bird_ID)*" specified in generalized linear mixed models (GLMMs). The response variables, sight rejection or attacking, represented each a binary outcome (sight rejected or not; actively attacked or not) using the family binomial (logit). Fixed effects included butterfly_type (type of butterfly; *Spicauda*, *Heliconius* or Euptychiina), state (bird age; juvenile or adult), and diet_reduced (diet category; insectivorous or other). The term (1|bird_ID) indicated a random effect for bird_ID (unique ID for each experimental bird), accounting for variability between individual birds.

Sight rejection behaviour

```
Family: binomial  ( logit )
Formula:          test_ignoring ~ butterfly_type + state + diet_reduced + (
1 |      bird_ID)
Data: data

     AIC      BIC   logLik deviance df.resid
   230.3    250.3   -109.2    218.3      202

Random effects:

Conditional model:
 Groups  Name        Variance Std.Dev.
 bird_ID (Intercept) 304.8    17.46
Number of obs: 208, groups:  bird_ID, 131

Conditional model:
                           Estimate Std. Error z value Pr(>|z|)
(Intercept)                 -7.9245     1.6288  -4.865 1.14e-06 ***
butterfly_typeHeliconius    14.7053     2.1245   6.922 4.46e-12 ***
butterfly_typeSpicauda      -0.1712     1.1547  -0.148    0.882
stateimmature                0.8342     1.1778   0.708    0.479
diet_reducedOther            0.3896     1.1968   0.326    0.745
---
Signif. codes:  0 '***' 0.001 '**' 0.01 '*' 0.05 '.' 0.1 ' ' 1
```

Emmeans results: butterfly type

```
$emmeans
 butterfly_type      prob       SE  df asymp.LCL asymp.UCL
 Euptychiina    0.0006666 0.000920 Inf 0.0000445  0.009903
 Heliconius     0.9993847 0.000713 Inf 0.9940578  0.999937
 Spicauda       0.0005618 0.000693 Inf 0.0000500  0.006280

Results are averaged over the levels of: state, diet_reduced
Confidence level used: 0.95
Intervals are back-transformed from the logit scale

$contrasts
 contrast                 odds.ratio       SE  df null z.ratio p.value
 Euptychiina / Heliconius   0.00e+00        0 Inf    1  -6.922  <.0001
 Euptychiina / Spicauda     1.20e+00        1 Inf    1   0.148  0.9880
 Heliconius / Spicauda      2.89e+06 5860000 Inf    1   7.333  <.0001

Results are averaged over the levels of: state, diet_reduced
P value adjustment: tukey method for comparing a family of 3 estimates
Tests are performed on the log odds ratio scale
```

Emmeans results: state (adult vs. immature)

```
$emmeans
 state      prob     SE  df asymp.LCL asymp.UCL
 adult    0.0529 0.0477 Inf   0.00856     0.266
 immature 0.1140 0.0879 Inf   0.02285     0.414

Results are averaged over the levels of: butterfly_type, diet_reduced
Confidence level used: 0.95
Intervals are back-transformed from the logit scale

$contrasts
 contrast         odds.ratio    SE  df null z.ratio p.value
 adult / immature      0.434 0.511 Inf    1  -0.708  0.4788

Results are averaged over the levels of: butterfly_type, diet_reduced
Tests are performed on the log odds ratio scale
```

Emmeans results: diet (insectivorous vs. other)

```
$emmeans
 diet_reduced    prob     SE  df asymp.LCL asymp.UCL
 Insectivorous 0.0652 0.0617 Inf    0.0095     0.337
 Other         0.0934 0.0689 Inf    0.0205     0.337

Results are averaged over the levels of: butterfly_type, state
Confidence level used: 0.95
Intervals are back-transformed from the logit scale

$contrasts
 contrast              odds.ratio    SE  df null z.ratio p.value
 Insectivorous / Other      0.677 0.811 Inf    1  -0.326  0.7447

Results are averaged over the levels of: butterfly_type, state
Tests are performed on the log odds ratio scale
```

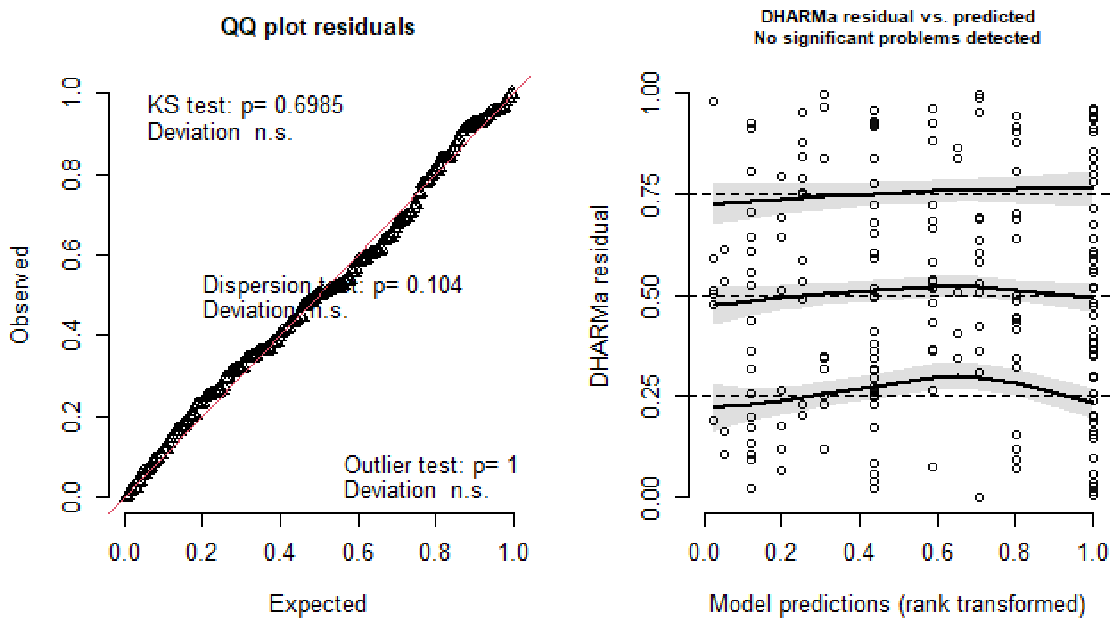

Figure S3: Diagnostic plots generated by the DHARMa package for the GLMM model testing sight rejection behaviour.

Attacking behaviour

```
Family: binomial  ( logit )
Formula:          test_attacking ~ butterfly_type + state + diet_reduced + 
(1 |      bird_ID)
Data: data

     AIC      BIC   logLik deviance df.resid 
   438.6    462.4   -213.3    426.6      387 

Random effects:

Conditional model:
 Groups  Name        Variance Std.Dev.
 bird_ID (Intercept) 0.8793   0.9377  
Number of obs: 393, groups:  bird_ID, 131

Conditional model:
                         Estimate Std. Error z value Pr(>|z|)    
(Intercept)               -1.5276     0.4160  -3.672 0.000241 ***
butterfly_typeHeliconius  -0.2758     0.3333  -0.827 0.407984    
butterfly_typeSpicauda     1.0421     0.3105   3.356 0.000791 ***
stateimmature             -0.1637     0.3271  -0.500 0.616807    
diet_reducedOther          0.1308     0.3379   0.387 0.698686    
---
Signif. codes:  0 '***' 0.001 '**' 0.01 '*' 0.05 '.' 0.1 ' ' 1
```

Emmeans results: butterfly type

```
$emmeans
 butterfly_type  prob     SE  df asymp.LCL asymp.UCL
 Euptychiina    0.176 0.0409 Inf    0.1093     0.271
 Heliconius     0.139 0.0362 Inf    0.0823     0.226
 Spicauda       0.377 0.0551 Inf    0.2765     0.489

Results are averaged over the levels of: state, diet_reduced 
Confidence level used: 0.95 
Intervals are back-transformed from the logit scale 

$contrasts
 contrast                 odds.ratio     SE  df null z.ratio p.value
 Euptychiina / Heliconius      1.318 0.4390 Inf    1   0.827  0.6860
 Euptychiina / Spicauda        0.353 0.1100 Inf    1  -3.356  0.0023
 Heliconius / Spicauda         0.268 0.0873 Inf    1  -4.040  0.0002

Results are averaged over the levels of: state, diet_reduced 
P value adjustment: tukey method for comparing a family of 3 estimates 
Tests are performed on the log odds ratio scale 
```

Emmeans results: state (adult vs. immature)

```
$emmeans
 state     prob     SE  df asymp.LCL asymp.UCL
 adult    0.230 0.0509 Inf     0.146     0.344
 immature 0.203 0.0368 Inf     0.140     0.284

Results are averaged over the levels of: butterfly_type, diet_reduced 
Confidence level used: 0.95 
Intervals are back-transformed from the logit scale 

$contrasts
 contrast         odds.ratio    SE  df null z.ratio p.value
 adult / immature       1.18 0.385 Inf    1   0.500  0.6168

Results are averaged over the levels of: butterfly_type, diet_reduced 
Tests are performed on the log odds ratio scale 
```

Emmeans results: diet (insectivorous vs. other)

```
$emmeans
 diet_reduced   prob     SE  df asymp.LCL asymp.UCL
 Insectivorous 0.205 0.0498 Inf     0.124     0.319
 Other         0.227 0.0372 Inf     0.163     0.308

Results are averaged over the levels of: butterfly_type, state
Confidence level used: 0.95
Intervals are back-transformed from the logit scale

$contrasts
 contrast              odds.ratio    SE  df null z.ratio p.value
 Insectivorous / Other      0.877 0.296 Inf    1  -0.387  0.6987

Results are averaged over the levels of: butterfly_type, state
Tests are performed on the log odds ratio scale
```

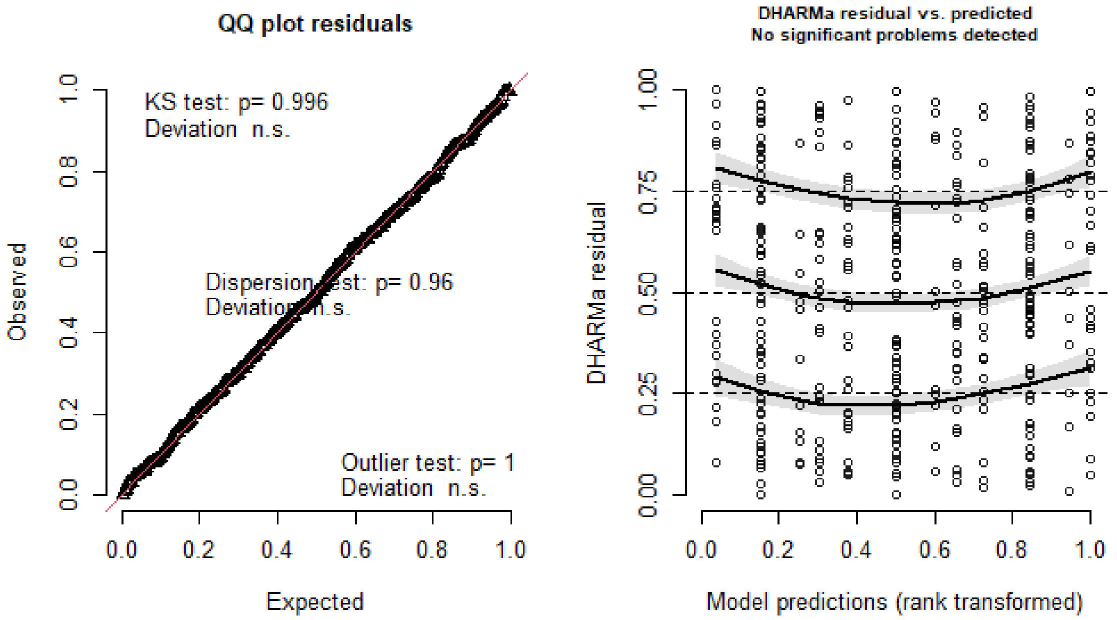

Figure S4: Diagnostic plots generated by the DHARMa package for the GLMM model testing attacking behaviour.

Additional GLMM models with habitat, season and bird family as fixed effects

The generalized linear mixed models (GLMMs) compared the likelihood of bird's sight rejection or attacking butterflies, using each a binomial distribution with a logit link. It included as fixed effects season (dry, wet), location (urban, forest), and bird family, while accounting for individual variation in birds as a random effect (1 | bird_ID). Bird families with less than five birds showing interactions with the butterflies were grouped together into "Other".

<u>Sight rejection behaviour</u>

```
Family: binomial  ( logit )
Formula:          test_ignoring ~ bird_family_reduced + season + location +
(1 |      bird_ID)
Data: data

     AIC      BIC   logLik deviance df.resid
   295.1    335.1   -135.5    271.1      196

Random effects:

Conditional model:
 Groups  Name        Variance Std.Dev.
 bird_ID (Intercept) 1.548    1.244
Number of obs: 208, groups:  bird_ID, 131

Conditional model:
                                 Estimate Std. Error z value Pr(>|z|)
(Intercept)                        1.7327     1.1153   1.554   0.1203
bird_family_reducedFurnariidae    -1.7033     1.2076  -1.411   0.1584
bird_family_reducedOther          -1.5886     1.4155  -1.122   0.2617
bird_family_reducedPipromorphidae -0.9279     1.1009  -0.843   0.3993
bird_family_reducedThamnophilidae -2.0931     1.2827  -1.632   0.1027
bird_family_reducedThraupidae     -2.0453     1.4701  -1.391   0.1641
bird_family_reducedTurdidae       -1.1424     1.0959  -1.042   0.2972
bird_family_reducedTyrannidae     -2.9467     1.5019  -1.962   0.0498 *
bird_family_reducedVireonidae     -3.2963     1.6265  -2.027   0.0427 *
seasonwet                         -0.4592     0.6548  -0.701   0.4832
locationTarapoto                   0.9621     0.9880   0.974   0.3302
---
Signif. codes:  0 '***' 0.001 '**' 0.01 '*' 0.05 '.' 0.1 ' ' 1
```

<u>Emmeans results: bird family</u>

```
$emmeans
 bird_family_reduced  prob    SE  df asymp.LCL asymp.UCL
 Cardinalidae        0.879 0.123 Inf    0.4292     0.986
 Furnariidae         0.570 0.181 Inf    0.2382     0.849
 Other               0.598 0.252 Inf    0.1597     0.921
 Pipromorphidae      0.742 0.124 Inf    0.4457     0.911
 Thamnophilidae      0.473 0.198 Inf    0.1589     0.810
 Thraupidae          0.485 0.186 Inf    0.1794     0.802
 Turdidae            0.699 0.169 Inf    0.3243     0.918
 Tyrannidae          0.276 0.148 Inf    0.0825     0.619
 Vireonidae          0.212 0.177 Inf    0.0328     0.681

Results are averaged over the levels of: season, location
Confidence level used: 0.95
Intervals are back-transformed from the logit scale
```

```
$contrasts
 contrast                          odds.ratio     SE  df null  z.ratio p.value
 Cardinalidae / Furnariidae             5.492  6.630 Inf    1   1.410  0.8942
 Cardinalidae / Other                   4.897  6.930 Inf    1   1.122  0.9710
 Cardinalidae / Pipromorphidae          2.529  2.780 Inf    1   0.843  0.9955
 Cardinalidae / Thamnophilidae          8.110 10.400 Inf    1   1.632  0.7874
 Cardinalidae / Thraupidae              7.731 11.400 Inf    1   1.391  0.9015
 Cardinalidae / Turdidae                3.134  3.430 Inf    1   1.042  0.9817
 Cardinalidae / Tyrannidae             19.043 28.600 Inf    1   1.962  0.5702
 Cardinalidae / Vireonidae             27.013 43.900 Inf    1   2.027  0.5245
 Furnariidae / Other                    0.892  1.090 Inf    1  -0.094  1.0000
 Furnariidae / Pipromorphidae           0.461  0.335 Inf    1  -1.066  0.9789
 Furnariidae / Thamnophilidae           1.477  1.320 Inf    1   0.437  1.0000
 Furnariidae / Thraupidae               1.408  1.690 Inf    1   0.285  1.0000
 Furnariidae / Turdidae                 0.571  0.531 Inf    1  -0.603  0.9996
 Furnariidae / Tyrannidae               3.467  4.220 Inf    1   1.021  0.9840
 Furnariidae / Vireonidae               4.918  6.840 Inf    1   1.146  0.9671
 Other / Pipromorphidae                 0.517  0.590 Inf    1  -0.579  0.9997
 Other / Thamnophilidae                 1.656  2.080 Inf    1   0.402  1.0000
 Other / Thraupidae                     1.579  2.080 Inf    1   0.347  1.0000
 Other / Turdidae                       0.640  0.719 Inf    1  -0.397  1.0000
 Other / Tyrannidae                     3.889  5.140 Inf    1   1.028  0.9833
 Other / Vireonidae                     5.517  8.150 Inf    1   1.156  0.9653
 Pipromorphidae / Thamnophilidae        3.207  2.600 Inf    1   1.439  0.8829
 Pipromorphidae / Thraupidae            3.057  3.530 Inf    1   0.967  0.9888
 Pipromorphidae / Turdidae              1.239  0.983 Inf    1   0.270  1.0000
 Pipromorphidae / Tyrannidae            7.529  8.920 Inf    1   1.705  0.7439
 Pipromorphidae / Vireonidae           10.680 14.500 Inf    1   1.748  0.7163
 Thamnophilidae / Thraupidae            0.953  1.190 Inf    1  -0.038  1.0000
 Thamnophilidae / Turdidae              0.386  0.385 Inf    1  -0.955  0.9897
 Thamnophilidae / Tyrannidae            2.348  2.930 Inf    1   0.685  0.9990
 Thamnophilidae / Vireonidae            3.331  4.650 Inf    1   0.862  0.9948
 Thraupidae / Turdidae                  0.405  0.477 Inf    1  -0.767  0.9977
 Thraupidae / Tyrannidae                2.463  2.120 Inf    1   1.046  0.9814
 Thraupidae / Vireonidae                3.494  4.180 Inf    1   1.046  0.9813
 Turdidae / Tyrannidae                  6.076  7.270 Inf    1   1.507  0.8525
 Turdidae / Vireonidae                  8.619 11.500 Inf    1   1.611  0.7990
 Tyrannidae / Vireonidae                1.419  1.650 Inf    1   0.301  1.0000

Results are averaged over the levels of: season, location
P value adjustment: tukey method for comparing a family of 9 estimates
Tests are performed on the log odds ratio scale
```

Emmeans results: season (dry vs. wet)

```
$emmeans
 season  prob     SE  df asymp.LCL asymp.UCL
 dry    0.614 0.1360 Inf     0.340     0.831
 wet    0.501 0.0827 Inf     0.345     0.658

Results are averaged over the levels of: bird_family_reduced, location
Confidence level used: 0.95
Intervals are back-transformed from the logit scale

$contrasts
 contrast  odds.ratio   SE  df null z.ratio p.value
 dry / wet       1.58 1.04 Inf    1   0.701  0.4832

Results are averaged over the levels of: bird_family_reduced, location
Tests are performed on the log odds ratio scale
```

Emmeans results: location (forested vs. urban)

```
$emmeans
 location      prob     SE  df asymp.LCL asymp.UCL
 Urban        0.439 0.0961 Inf     0.267     0.627
 Forested     0.672 0.1650 Inf     0.320     0.899

Results are averaged over the levels of: bird_family_reduced, season
Confidence level used: 0.95
Intervals are back-transformed from the logit scale

$contrasts
 contrast              odds.ratio    SE  df null z.ratio p.value
 Forested / Urban       0.382 0.378 Inf    1  -0.974  0.3302

Results are averaged over the levels of: bird_family_reduced, season
Tests are performed on the log odds ratio scale
```

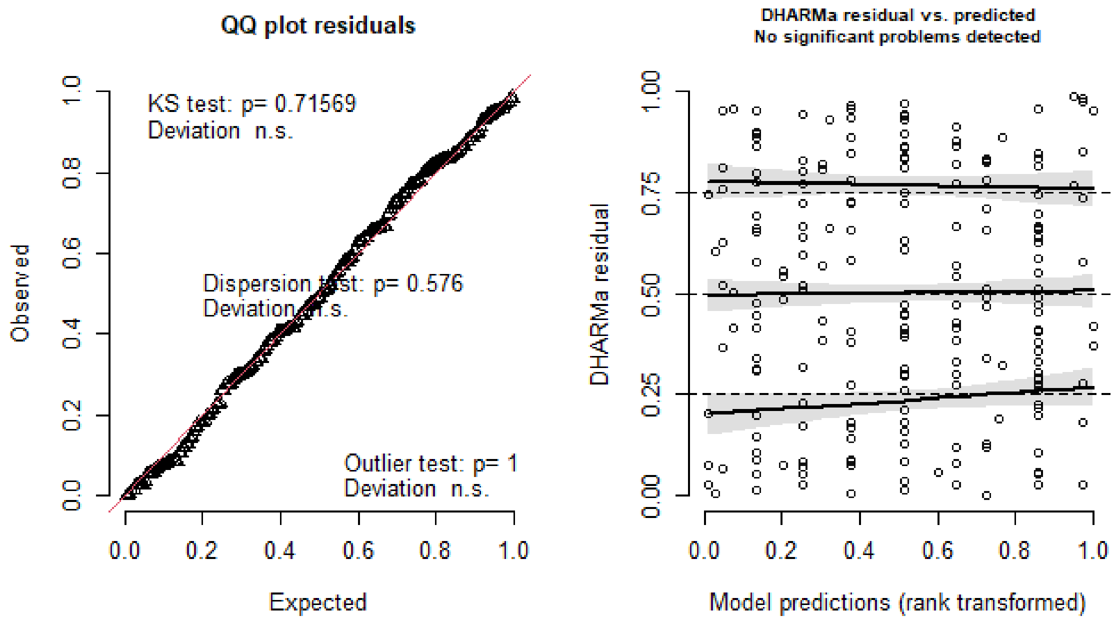

Figure S5: Diagnostic plots generated by the DHARMa package for the additional GLMM model testing sight rejection behaviour.

Attacking behaviour

```
Family: binomial  ( logit )
Formula:         test_attacking ~ bird_family_reduced + season + location
+ (1 |      bird_ID)
Data: data

     AIC      BIC   logLik deviance df.resid
   461.3    508.9   -218.6    437.3      381

Random effects:

Conditional model:
 Groups  Name        Variance Std.Dev.
 bird_ID (Intercept) 0.418    0.6465
Number of obs: 393, groups:  bird_ID, 131

Conditional model:
                                  Estimate Std. Error z value Pr(>|z|)
(Intercept)                        -2.0547     0.7903  -2.600  0.00933 **
bird_family_reducedFurnariidae      1.2670     0.8477   1.495  0.13502
bird_family_reducedOther            0.6682     0.9774   0.684  0.49416
bird_family_reducedPipromorphidae   0.3928     0.7965   0.493  0.62189
bird_family_reducedThamnophilidae   0.7393     0.8462   0.874  0.38229
bird_family_reducedThraupidae       1.5345     0.9967   1.540  0.12368
bird_family_reducedTurdidae         0.7029     0.7899   0.890  0.37354
bird_family_reducedTyrannidae       1.8480     0.9766   1.892  0.05846 .
bird_family_reducedVireonidae       2.1287     1.0051   2.118  0.03418 *
seasonwet                           0.4202     0.4305   0.976  0.32906
locationTarapoto                   -1.1164     0.6235  -1.790  0.07338 .
---
Signif. codes:  0 ‘***’ 0.001 ‘**’ 0.01 ‘*’ 0.05 ‘.’ 0.1 ‘ ’ 1
```

Emmeans results: bird family

```
$emmeans
 bird_family_reduced  prob     SE  df asymp.LCL asymp.UCL
 Cardinalidae        0.083 0.0617 Inf    0.0181     0.307
 Furnariidae         0.243 0.0872 Inf    0.1125     0.449
 Other               0.150 0.0896 Inf    0.0426     0.411
 Pipromorphidae      0.118 0.0448 Inf    0.0545     0.237
 Thamnophilidae      0.159 0.0675 Inf    0.0659     0.337
 Thraupidae          0.296 0.1040 Inf    0.1362     0.528
 Turdidae            0.154 0.0693 Inf    0.0606     0.341
 Tyrannidae          0.365 0.1040 Inf    0.1926     0.580
 Vireonidae          0.432 0.1500 Inf    0.1860     0.717

Results are averaged over the levels of: season, location
Confidence level used: 0.95
Intervals are back-transformed from the logit scale

$contrasts
 contrast                        odds.ratio    SE  df null z.ratio p.value
 Cardinalidae / Furnariidae           0.282 0.239 Inf    1  -1.495  0.8585
 Cardinalidae / Other                 0.513 0.501 Inf    1  -0.684  0.9990
 Cardinalidae / Pipromorphidae        0.675 0.538 Inf    1  -0.493  0.9999
 Cardinalidae / Thamnophilidae        0.477 0.404 Inf    1  -0.874  0.9943
 Cardinalidae / Thraupidae            0.216 0.215 Inf    1  -1.540  0.8369
 Cardinalidae / Turdidae              0.495 0.391 Inf    1  -0.890  0.9935
 Cardinalidae / Tyrannidae            0.158 0.154 Inf    1  -1.892  0.6192
 Cardinalidae / Vireonidae            0.119 0.120 Inf    1  -2.118  0.4608
 Furnariidae / Other                  1.820 1.450 Inf    1   0.749  0.9980
 Furnariidae / Pipromorphidae         2.397 1.170 Inf    1   1.794  0.6866
 Furnariidae / Thamnophilidae         1.695 0.944 Inf    1   0.947  0.9902
 Furnariidae / Thraupidae             0.765 0.592 Inf    1  -0.346  1.0000
 Furnariidae / Turdidae               1.758 1.070 Inf    1   0.923  0.9918
 Furnariidae / Tyrannidae             0.559 0.417 Inf    1  -0.779  0.9974
 Furnariidae / Vireonidae             0.422 0.343 Inf    1  -1.060  0.9796
 Other / Pipromorphidae               1.317 0.996 Inf    1   0.364  1.0000
```

```
 Other / Thamnophilidae                 0.931 0.750 Inf    1  -0.088  1.0000
 Other / Thraupidae                     0.421 0.369 Inf    1  -0.987  0.9871
 Other / Turdidae                       0.966 0.722 Inf    1  -0.046  1.0000
 Other / Tyrannidae                     0.307 0.262 Inf    1  -1.385  0.9038
 Other / Vireonidae                     0.232 0.211 Inf    1  -1.607  0.8016
 Pipromorphidae / Thamnophilidae        0.707 0.348 Inf    1  -0.704  0.9987
 Pipromorphidae / Thraupidae            0.319 0.240 Inf    1  -1.517  0.8481
 Pipromorphidae / Turdidae              0.733 0.396 Inf    1  -0.575  0.9997
 Pipromorphidae / Tyrannidae            0.233 0.169 Inf    1  -2.003  0.5409
 Pipromorphidae / Vireonidae            0.176 0.139 Inf    1  -2.208  0.4001
 Thamnophilidae / Thraupidae            0.452 0.361 Inf    1  -0.994  0.9866
 Thamnophilidae / Turdidae              1.037 0.633 Inf    1   0.060  1.0000
 Thamnophilidae / Tyrannidae            0.330 0.256 Inf    1  -1.431  0.8862
 Thamnophilidae / Vireonidae            0.249 0.207 Inf    1  -1.671  0.7642
 Thraupidae / Turdidae                  2.297 1.760 Inf    1   1.085  0.9765
 Thraupidae / Tyrannidae                0.731 0.402 Inf    1  -0.570  0.9997
 Thraupidae / Vireonidae                0.552 0.406 Inf    1  -0.807  0.9967
 Turdidae / Tyrannidae                  0.318 0.235 Inf    1  -1.551  0.8313
 Turdidae / Vireonidae                  0.240 0.185 Inf    1  -1.851  0.6479
 Tyrannidae / Vireonidae                0.755 0.529 Inf    1  -0.401  1.0000

Results are averaged over the levels of: season, location
P value adjustment: tukey method for comparing a family of 9 estimates
Tests are performed on the log odds ratio scale
```

<u>Emmeans results: season (dry vs. wet)</u>

```
$emmeans
 season  prob     SE  df asymp.LCL asymp.UCL
 dry    0.171 0.0555 Inf    0.0871     0.307
 wet    0.238 0.0417 Inf    0.1664     0.329

Results are averaged over the levels of: bird_family_reduced, location
Confidence level used: 0.95
Intervals are back-transformed from the logit scale

$contrasts
 contrast  odds.ratio    SE  df null z.ratio p.value
 dry / wet      0.657 0.283 Inf    1  -0.976  0.3291

Results are averaged over the levels of: bird_family_reduced, location
Tests are performed on the log odds ratio scale
```

<u>Emmeans results: location (forested vs. urban)</u>

```
$emmeans
 location  prob     SE  df asymp.LCL asymp.UCL
 Chacra   0.307 0.0537 Inf    0.2129     0.421
 Tarapoto 0.127 0.0547 Inf    0.0522     0.277

Results are averaged over the levels of: bird_family_reduced, season
Confidence level used: 0.95
Intervals are back-transformed from the logit scale

$contrasts
 contrast          odds.ratio  SE  df null z.ratio p.value
 Chacra / Tarapoto       3.05 1.9 Inf    1   1.790  0.0734

Results are averaged over the levels of: bird_family_reduced, season
Tests are performed on the log odds ratio scale
```

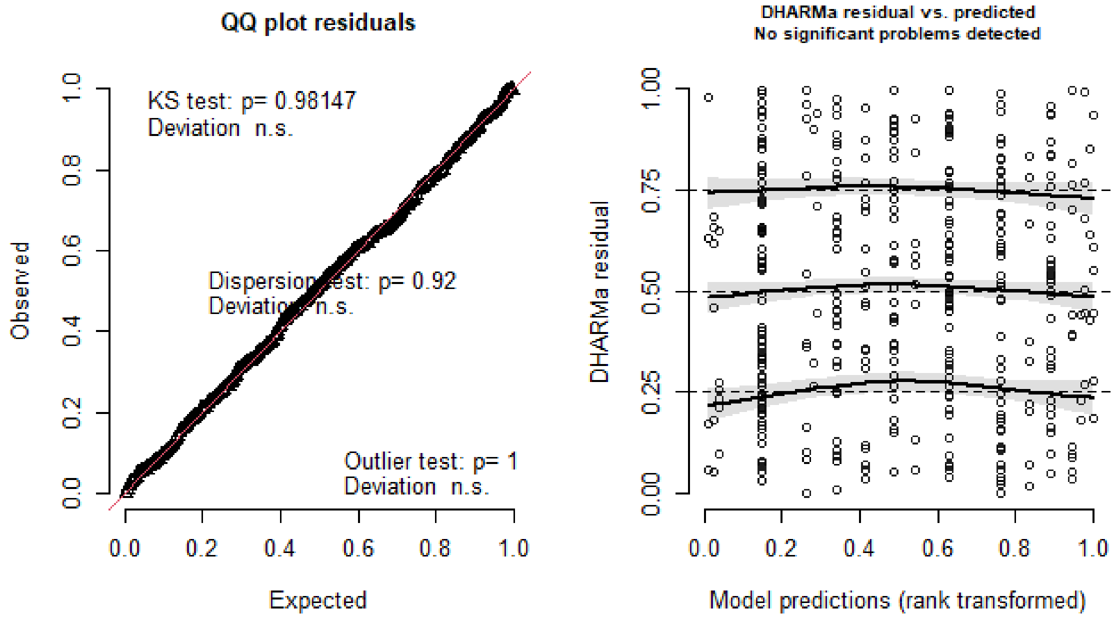

Figure S6: Diagnostic plots generated by the DHARMa package for the additional GLMM model testing attacking behaviour.

Section S6: Stratum selection in the aviaries (high > 200 cm, low < 200 cm) by bird families a nd butterfly type.

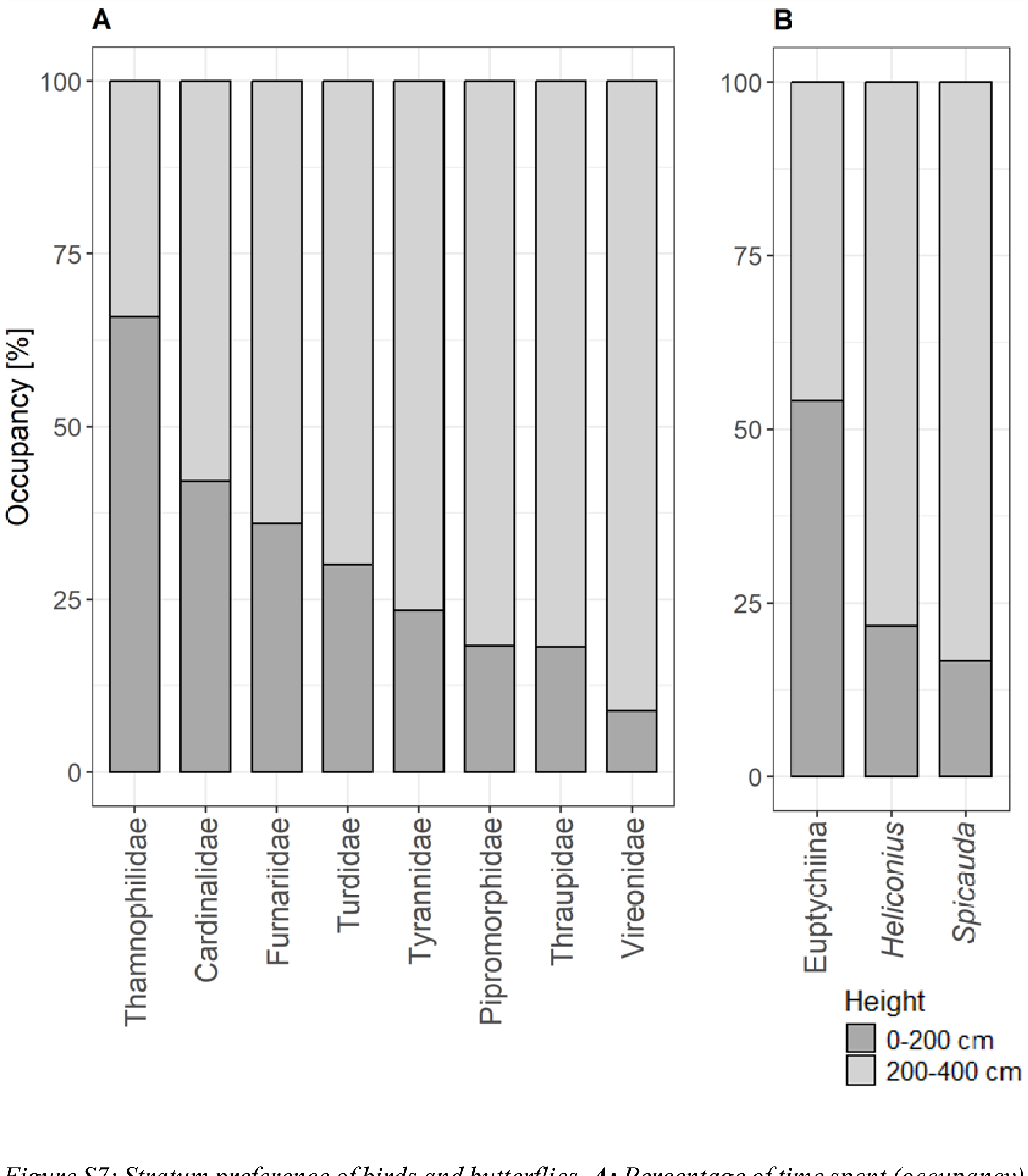

Figure S7: Stratum preference of birds and butterflies. ***A:*** *Percentage of time spent (occupancy) by birds in the bottom, 0–200cm and the top, 200–400cm, of the aviary during the experiment (60 minutes). In the graph, only bird families with at least 5 valid experiments are presented.* ***B:*** *Percentage of time spent by the three prey phenotypes (Euptychiines, Heliconius and Spicauda) at the two different heights in the aviary.*

Section S7: Results of log-likelihood tests for habitat and season.

Table S4: Likelihood-based model tests of scenarios depicting the effectiveness of antipredator defences by three butterfly prey along the predation sequence. Akaike weights for the total number of birds, season (dry vs. wet) and habitat (urban vs. forest) are shown. Best models are marked in ***bold and underlined****, while competing models (ΔAICc < 2) are only* ***bold****. Values in brackets represent ΔAICc values compared to the best performing model. Sample sizes for (1) to (3.2) are identical for all prey types: 131 for total, 37 for urban, 94 for forest, 54 for dry season and 77 for wet season. For (4) and (5) sample sizes are different per prey type (depending on how many birds choose to attack each butterfly type) for the total, Heliconius has a sample size of 23, Spicauda of 52 and Euptychiines of 28, for urban 1,21 and 8, forest 22,31 and 20, dry season 13,15 and 9 and wet season 10,37 and 19, respectively.*

**Stage**	**Model**	**All different**	**All equal**	***Heliconius* different**	***Spicauda* different**	**Euptychiina different**
(1) Remaining undetected	Total	**0.47** (0.18)	0.00 (15.45)	0.02 (6.98)	0.00 (16.58)	**0.51** (0.00)
	Urban	**0.15** (1.25)	**0.22** (0.51)	0.08 (2.58)	**0.28** (0.02)	**0.28** (0.00)
	Forest	**0.72** (0.00)	0.00 (16.55)	0.13 (3.47)	0.00 (18.56)	0.15 (3.10)
	Dry season	**0.53** (0.00)	0.00 (12.65)	**0.41** (0.50)	0.00 (14.44)	0.06 (4.42)
	Wet season	**0.24** (1.94)	0.04 (5.70)	0.02 (6.50)	0.05 (4.93)	**0.64** (0.00)
(2) Rejection upon identification	Total	**0.50** (0.00)	0.00 (26.34)	**0.50** (0.02)	0.00 (25.15)	0.00 (13.10)
	Urban	0.23 (2.04)	0.03 (5.97)	**0.65** (0.00)	0.05 (5.09)	0.03 (6.22)
	Forest	**0.62** (0.00)	0.00 (19.54)	**0.37** (1.04)	0.00 (20.32)	0.01 (7.53)
	Dry season	**0.40** (0.76)	0.00 (12.91)	**0.58** (0.00)	0.00 (13.39)	0.02 (6.49)
	Wet season	**0.34** (1.21)	0.00 (11.68)	**0.63** (0.00)	0.00 (12.06)	0.02 (6.64)
(3.1) Targeting probability	Total	**0.25** (1.77)	0.04 (5.73)	0.07 (4.29)	**0.62** (0.00)	0.02 (6.70)
	Urban	**0.50** (0.00)	0.00 (9.32)	0.17 (2.16)	**0.32** (0.91)	0.00 (11.03)
	Forest	0.10 (2.45)	**0.33** (0.00)	**0.13** (1.83)	**0.26** (0.46)	**0.17** (1.34)
	Dry season	0.08 (3.25)	**0.39** (0.00)	0.14 (2.02)	**0.18** (1.57)	**0.21** (1.26)
	Wet season	**0.36** (0.54)	0.01 (6.99)	0.14 (2.49)	**0.48** (0.00)	0.01 (8.64)
(3.2) Capture avoidance	Total	0.09 (2.89)	**0.37** (0.00)	**0.16** (1.66)	**0.14** (1.88)	**0.24** (0.86)
	Urban	0.06 (3.77)	**0.42** (0.00)	**0.18** (1.62)	**0.18** (1.67)	**0.15** (2.00)
	Forest	0.08 (3.23)	**0.39** (0.00)	**0.17** (1.62)	0.14 (2.06)	**0.22** (1.17)
	Dry season	0.09 (2.80)	**0.35** (0.00)	0.13 (2.05)	**0.15** (1.64)	**0.28** (0.45)
	Wet season	0.06 (4.13)	**0.45** (0.00)	0.16 (2.03)	0.16 (2.11)	**0.17** (1.98)

(4) Surviving attack	Total	0.06 (3.79)	**0.43** (0.00)	**0.16** (1.99)	**0.16** (1.97)	**0.19** (1.67)
	Urban	0.06 (3.96)	**0.44** (0.00)	**0.21** (1.48)	0.14 (2.26)	0.14 (2.29)
	Forest	0.07 (3.32)	**0.39** (0.00)	0.14 (2.11)	**0.19** (1.50)	**0.21** (1.27)
	Dry season	0.06 (4.20)	**0.45** (0.00)	**0.17** (1.97)	0.15 (2.24)	**0.17** (1.93)
	Wet season	0.06 (3.93)	**0.43** (0.00)	**0.16** (1.97)	**0.18** (1.74)	**0.16** (2.00)

Section S8: Outcomes of predation experiments depending on habitat (Forest vs. Urban) season (Dry vs. Wet)

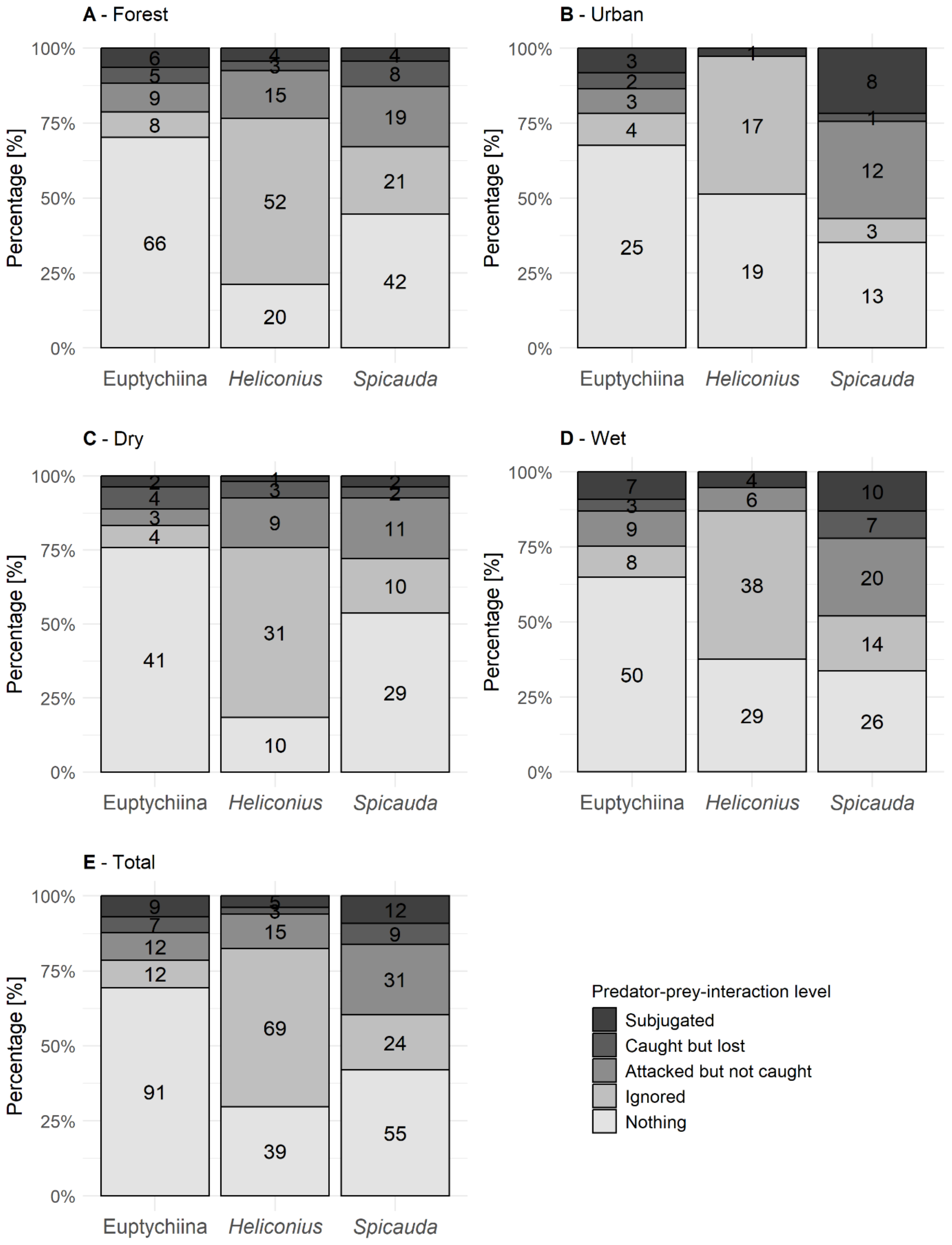

Figure S8: Comparison of predation outcomes (nothing, sight rejected after detection, attacked, caught or subjugated) depending on the butterfly type (Euptychiines, Heliconius and Spicauda),

season (dry and wet) and habitat (urban or forest). Numbers inside bars represents number of birds showing this interaction. ***A****: distribution for birds caught in the forested environment;* ***B****: distribution for all birds from the urban environment;* ***C****: distribution for all birds caught during the dry season;* ***D****: distribution for all birds caught during the wet season and* ***E****: distribution of the total dataset for comparison. Only the highest observed predation stage per bird-butterfly pair was used. Numbers inside bars indicate sample size.*
